# Direct alkenylation of indolin-2-ones by 6-aryl-4-methylthio-2*H*-pyran-2-one-3-carbonitriles: a novel approach

**DOI:** 10.3762/bjoc.9.92

**Published:** 2013-04-25

**Authors:** Sandeep Kumar, Ramendra Pratap, Abhinav Kumar, Brijesh Kumar, Vishnu K Tandon, Vishnu Ji Ram

**Affiliations:** 1 Department of Chemistry, University of Lucknow, Lucknow-226007, India; 2 Department of Chemistry, North Campus, University of Delhi, New Delhi-110007, India; 3 Department of SAIF, Central Drug Research Institute, Lucknow-226001, India

**Keywords:** alkenylation, dibenzo[*d,f*][1,3]diazepin-6(7*H*)-one, indolin-2-one, ketene dithioacetal, 2*H*-pyran-2-one

## Abstract

A direct one-pot base-induced alkenylation of indolin-2-ones has been developed by using 6-aryl-4-methylthio-2*H*-pyran-2-one-3-carbonitriles. Different bases such as MeONa, NaH and *t*-BuONa have been used to optimize the reaction conditions to obtain the desired product. NaH in THF was found to be the most suitable for the alkenylation of indolin-2-ones. Reaction in the presence of other bases led to the formation of 1-aryl-3-methoxy/methylthio-5*H*-dibenzo[*d,f*][1,3]diazepin-6(7*H*)-ones. Quantum chemical calculations have been performed to explain the nature of the weak noncovalent interactions operating in the supramolecular architectures of alkenylated indoline-2-ones and to explain the relative stability of one of the tautomers with respect to the others.

## Introduction

6-Aryl-4-methylthio-2*H*-pyran-2-one-3-carbonitriles have emerged as versatile synthons for the construction of an array of arenes and heteroarenes through base-induced ring transformation by nitrogen, sulfur and carbon nucleophiles [1]. However, suitably functionalized 2*H*-pyran-2-ones have not been investigated for the alkenylation of indolin-2-ones. An extensive literature survey on the pharmacological properties of 3-alkenylindolin-2-ones revealed that they possess potent antitumor [2–5], antipyretic [6], antifungal [7–8], anti-inflammatory [9], and analgesic [9] activities. In addition, they also act as inhibitors of lipoxygenase and butyrylcholinesterase enzymes [10].

While alkylations and arylations of indole are well documented in the literature [11–19], acid-catalyzed alkenylation by α-oxo ketene dithioacetals [20] have only recently been reported. There are plenty of literature reports available on the construction of 3-alkenylindolin-2-ones [21]. The widely used highly facile protocol for the alkenylation of indolin-2-ones is through aldol condensation [22] of isatin with compounds containing an active methylene group as well as by Wittig reaction [23–24]. The growing importance of 3-alkenylindolin-2-ones has resulted in the design of numerous new synthetic routes. Recently, metal-catalyzed carbonylative annulation of alkynyl-arylamines has been employed for the synthesis of this class of compounds [25–31]. More recently, Kamijo, Yamamoto and co-workers [32] have developed a palladium-catalyzed cyclization of acetylenic aryl isocyanates in the presence of terminal alkynes. Halogenated arylpropionamides are commonly employed for the preparation of 3-alkenylindolin-2-ones involving tin hydride-AIBN initiated radical cyclization [33–34]. In 2005, Player and co-workers reported a tandem Heck/Suzuki–Miyaura coupling process for the synthesis of (*E*)-3,3-(diaryl)oxindoles [35–37]. Recently, alkenylation of indolin-2-ones has been developed by palladium-catalyzed aromatic C–H activation/Heck reaction starting from *N*-acryloylanilides [38]. The use of metal catalysis, especially of palladium, has played a major role in the construction of 3-alkenylindolin-2-ones [38]. Despite significant progress in approaches for the construction of 3-alkenylindolin-2-ones, use of complicated precursors, expensive and sensitive metal catalysts, harsh reaction conditions and incompatibility of reagents towards the functional groups, restricted their frequent application. Thus, development of new efficient routes by direct alkenylation, free from the shortcomings of past procedures, is highly demanding and remains a challenge to the state-of-art synthesis. The promising pharmacological activities of 3-alkenylindolin-2-ones, prompted us to develop an efficient and concise route for their construction. Based on the topography and electronic features of 6-aryl-4-methylthio-2*H*-pyran-2-one-3-carbonitriles **3**, we envisioned their use to alkenylate indolin-2-ones to deliver 3-alkenylindolin-2-ones. An extensive literature survey revealed that 6-aryl-4-methylthio-2*H*-pyran-2-one-3-carbonitriles **3** have never been employed to alkenylate indolin-2-ones.

## Results and Discussion

### Synthesis

**Scheme 1:**
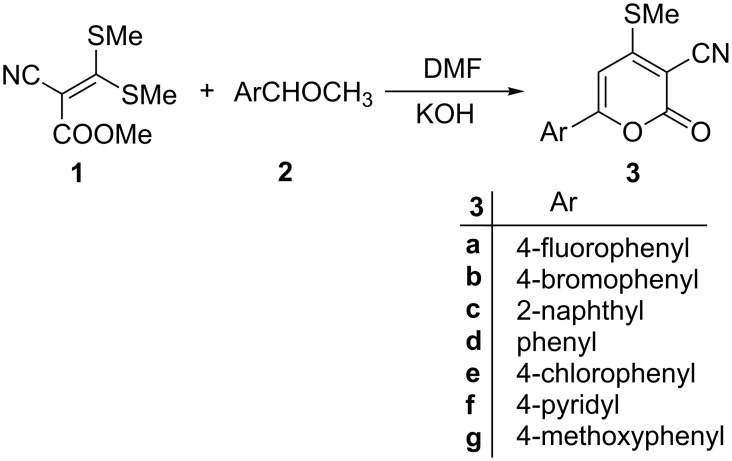
Syntheses of 6-aryl-4-methylthio-2*H*-pyran-2-one-3-carbonitriles **3**.

Herein, we report a short and efficient approach for the alkenylation of indolin-2-ones by suitably functionalized 6-aryl-4-methylthio-2*H*-pyran-2-one-3-carbonitriles **3**. The precursor **3** was prepared from the reaction of methyl 2-cyano-3,3-dimethylthioacrylate [39–40] (**1**) and aryl methyl ketone (**2**) in the presence of powdered KOH in DMF at room temperature as reported earlier [41–42] (Scheme 1).

**Figure 1:**
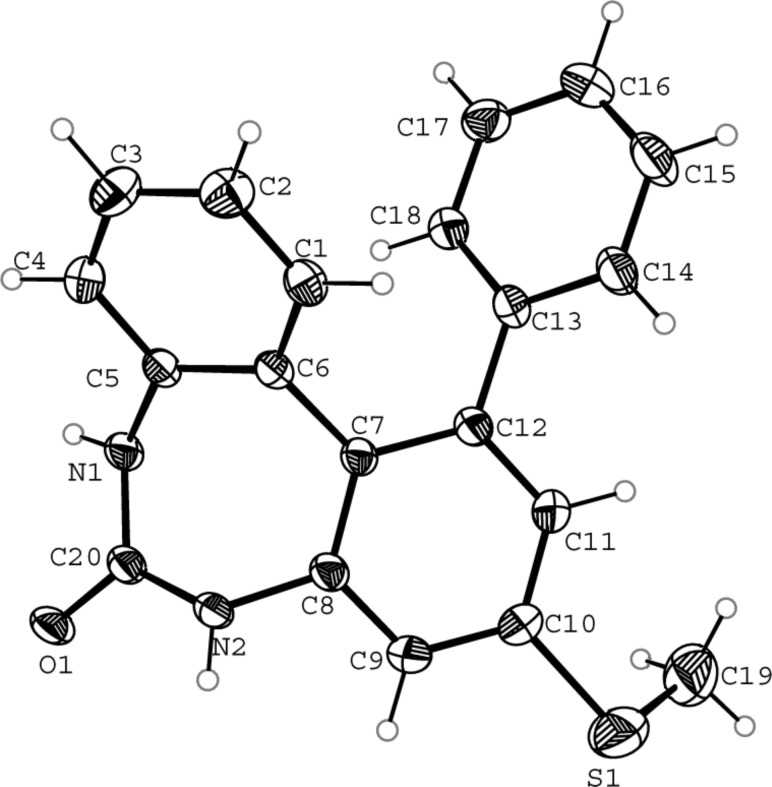
ORTEP view with atom numbering scheme of compound **5** with displacement ellipsoids at the 30% probability level.

**Scheme 2:**
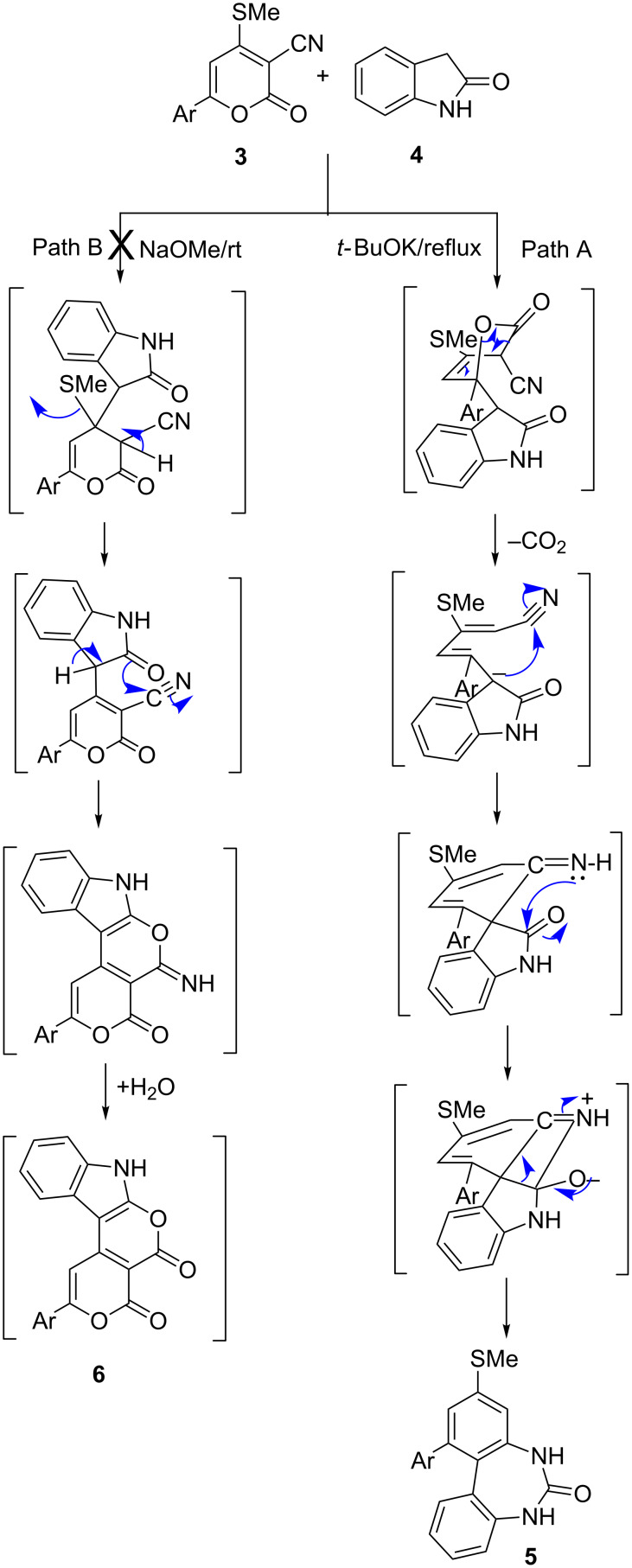
A plausible mechanism for the formation of 1-aryl-3-methylthio-5*H*-dibenzo[*d,f*][1,3]diazepin-6(7*H*)-ones.

**Scheme 3:**
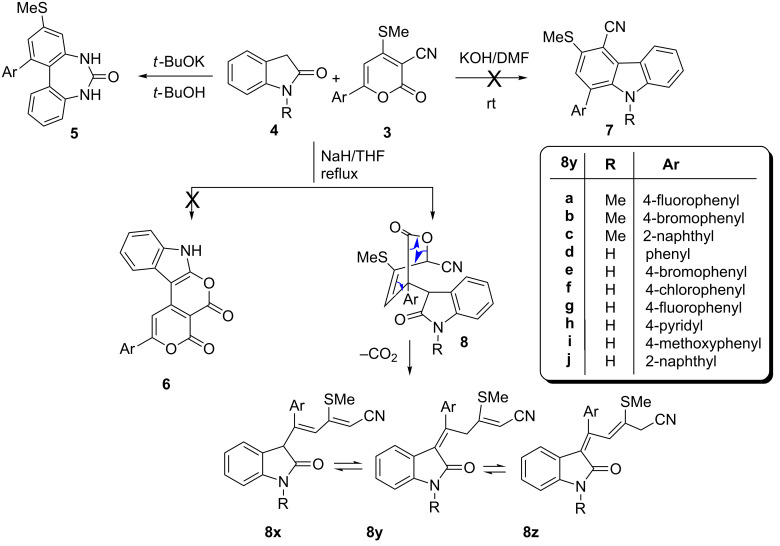
Synthesis of 3-alkenylindolin-2-ones.

The reaction between indolin-2-one (**4**) and 6-aryl-4-methylthio-2*H*-pyran-2-one-3-carbonitriles **3** in the presence of *t*-BuOK/MeONa in *tert*-butanol/methanol under reflux resulted in 1-aryl-3-methylthio-5*H*-dibenzo[*d,f*][1,3]diazepin-6(7*H*)-ones (**5**, Figure 1) instead of the mechanistically possible products 2-phenyl-3,6-dioxa-4,5-dioxonaphtho[2,1-*b*]-7*H*-indole **6** or a carbazole derivative **7** or 3-alkenylindolin-2-ones **8** (Scheme 2 and Scheme 3).

Further, reactions of **3** and **4** in the presence of NaH as a base in THF under reflux gave a product entirely different from **5, 6** and **7**. The isolated product was finally characterized by single-crystal X-ray diffraction as (2*Z,*5*E*)-5-aryl-3-methylthio-5-(2-oxoindolin-3-ylidene)pent-2-enenitrile **8**. The reaction is initiated with Michael addition at C6 followed by ring opening with elimination of carbon dioxide to deliver product **8** without undergoing further cyclization to yield either **6** or **7**, possibly due to the lower dielectric constant of THF (7.42) compared to methanol (32.7) (Scheme 3).

**Figure 2:**
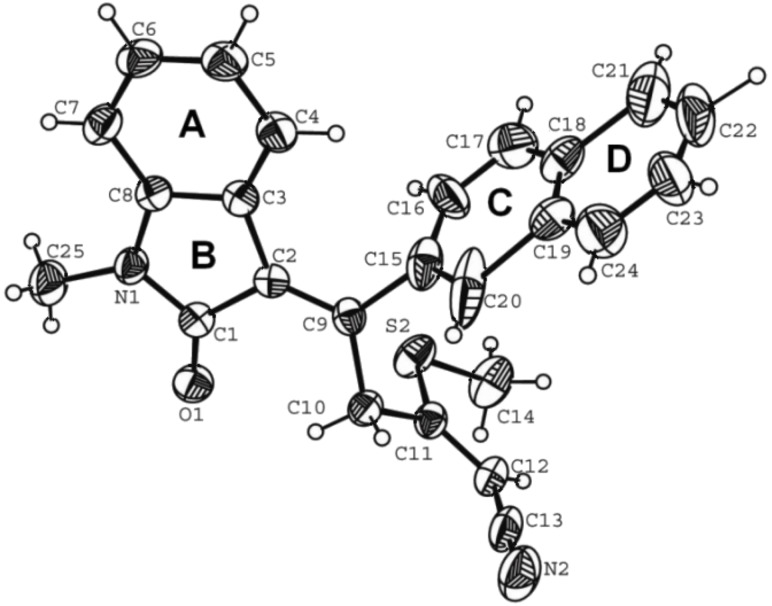
ORTEP view with atom numbering scheme of compound **8yc** with displacement ellipsoids at the 30% probability level.

As is evident from Scheme 3, there are three possible tautomeric forms for **8**, viz. **8x**, **8y**, **8z** for the isolated compound. Single-crystal X-ray studies revealed that out of the three tautomeric forms, the **8y** is the most suitable structure based on the bond lengths. Quantum chemical calculations have been performed in order to gain information regarding the relative energy difference, which in turn reflects the relative stability between the tautomeric forms **8x**, **8y** and **8z**. The energy calculations at the DFT level of theory for all of the three tautomers indicate that **8y** is 17.07 kJ·mol^−1^ more stable than **8x**, and it is more stable than **8z** by 14.84 kJ·mol^−1^. A deep structural study of **8y** indicates the formation of intramolecular C–H^…^O interaction which may be responsible for its extra stability compared to **8x** and **8z**. However, for **8z** the formation of intramolecular C–H^…^O interactions is also possible, but the existence of the relatively more stable *trans*-**8z** nullifies the likelihood of this type of interaction. Hence, density functional theory (DFT) calculations also indicate that the tautomer **8y** is relatively more stable than **8x** and **8z** (Figure 2).

**Scheme 4:**
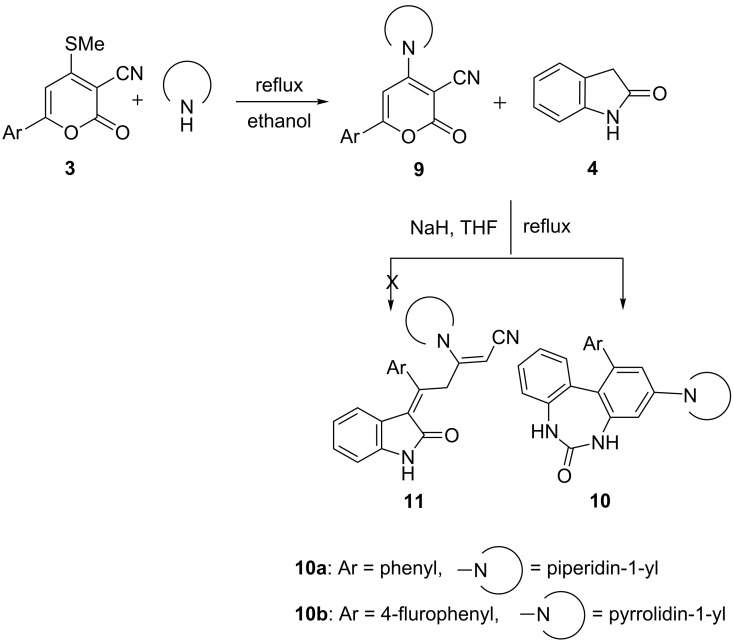
Synthesis of 1-aryl-3-*sec*-amino-5*H*-dibenzo[*d,f*][1,3]diazepin-6(7*H*)-ones **10**.

Further, in order to generalize the reaction, attempts were made for the alkenylation of indolin-2-one with 6-aryl-4-*sec*-amino-2*H*-pyran-2-one-3-carbonitriles **9** [41–42], obtained by the amination of **3** with *sec*-amine in boiling ethanol to yield a *sec*-amino substituted alkenylated chain on position 3 of the indolin-2-one (**4**). But to our utmost surprise the expected alkenylated product **11** could not be isolated. However, in lieu of this, the product isolated was characterized as 1-aryl-3-*sec*-amino-5*H*-dibenzo[*d,f*][1,3]diazepin-6(7*H*)-one **10**. The formation of **10** is possible only if the reaction is initiated with Michael addition of indolin-2-one at C6 not at C4, due to the presence of the secondary amino group, which reduces its electrophilicity. Thus, preferential attack by carbanion generated from **4** at C6 was inevitable for the formation of **10** (Scheme 4).

### X-ray crystallography

The molecular view (ORTEP) for the compounds **8yc** with its atom numbering scheme is presented in Figure 2. The compound **8yc** crystallizes in a monoclinic crystal system having *P*2_1_/*c* space group with four molecules in the unit cell. The rings A and B are coplanar with respect to each other. However, the dihedral angle between the rings A and C is 73.52°. The bond lengths C2–C9, C9–C10, C9–C15, C10–C11 and C11–C12 have dimensions 1.348(5), 1.513(5), 1.501(7), 1.510(5) and 1.331(5) Å, respectively. The angles <C2–C9–C15, <C2–C11–C12 have magnitudes of 119.7(4)°, 123.2(3)° and 123.2(4)°, respectively.

**Figure 3:**
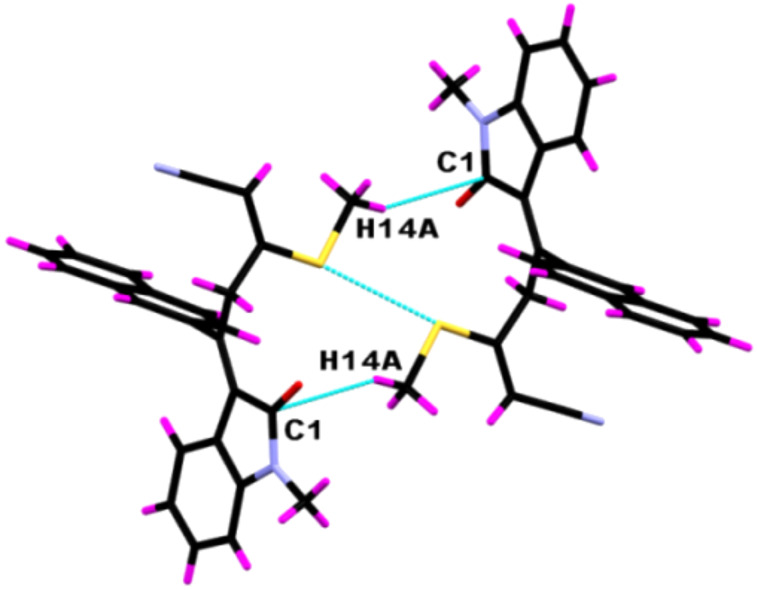
Centrosymmetric dimer of **8yc** bound by a pair of weak C−H^…^π intermolecular interactions (symm. op. 2 − *x*,1 − *y*, 2 − *z*).

**Figure 4:**
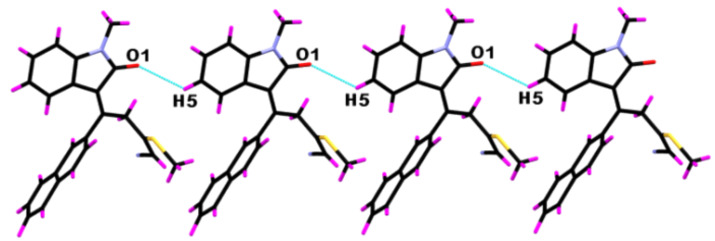
Supramolecular chain of **8yc** bound by weak C−H^…^O intermolecular interactions (symm. op. *x*,1 + *y*, *z*).

**Figure 5:**
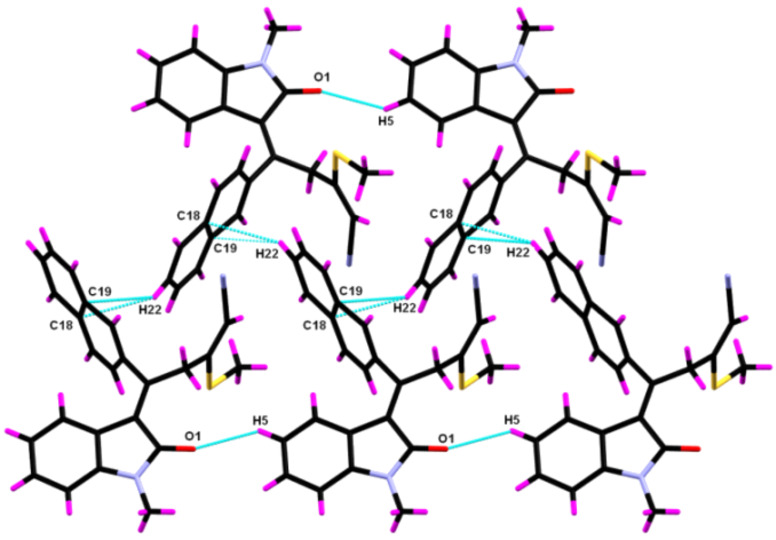
Supramolecular chain of **8yc** bound by weak C−H^…^O and Ar-H^…^π intermolecular interactions (symm. op. 2 − *x*,−1/2 + *y*,3/2 − *z*).

The supramolecular aggregations in **8yc** are stabilized by a pair of weak C−H^…^π interactions (Figure 3) that led to the formation of a centrosymmetric dimer. The C–H^…^π interaction distance is 2.883 Å and the angle <C–H^…^π is 148.97°. Additionally, the C5–H5^…^O1 intermolecular interactions lead to the formation of a molecular chain having a H5^…^O1 interaction length of 2.716 Å and a C5–H5^…^O1 interaction angle of 127.60° (Figure 4). Along with the C–H^…^O interactions the molecule displays Ar–H^…^π interactions (Figure 5) having H22^…^C18 and H22^…^C19 interaction distances of 2.783 and 2.794 Å, respectively.

In order to analyze the various interactions that lead to the crystal structure, interaction energies and electrostatic potentials were calculated for dimer fragments (Figure 5). The analysis of the interaction energy in the crystal structures of **8yc** by means of the dimer unit bound by C–H^…^π, C–H^…^O and Ar–H^…^π interactions at the DFT level of theory yields interaction energies of 22.33, 14.92 and 15.45 kJ·mol^−1^, respectively. To confirm further the presence of these weak interactions, bond critical points (bcp) were calculated for the different dimers by using the atoms in molecules theory [43]. The bond critical points observed between the interacting atoms confirm the presence of weak noncovalent interactions between the two molecules of **8yc**. The value of electron density (ρ), Laplacian of the electron density (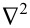
ρ_bcp_), bond ellipticity (ε), electron density (ρ), and total energy density (H) at the bond critical point for all the three interactions are presented in Table 1. As indicated in Table 1, the electron densities for all the three types of interactions at the bond critical point (ρ_bcp_) are less than +0.10 au, which indicates closed-shell hydrogen bonding interactions. Additionally, the Laplacians of the electron density 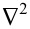
ρ_bcp_ in all the three cases are greater than zero, which indicates the depletion of electron density in the region of contact between the H^…^O and H^…^C atoms. The bond ellipticity (ε) measures the extent to which the density is preferentially accumulated in a given plane containing the bond path. The ε values for all the three interactions indicate that these are not cylindrically symmetrical in nature.

**Table 1:** Selected topographical features for various interactions computed at the B3LYP/6-31G** level of theory.

Interaction type	ρ_bcp_	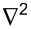 ρ_bcp_	ε	H (au)

C–H^…^π	+0.016942	+0.072948	+0.019047	+0.003173
C–O^…^H	+0.008118	+0.044151	+0.221799	+0.050545
Ar–H^…^π	+0.008257	+0.031372	+0.224819	+0.003505

## Conclusion

Owing to the numerous procedures known for the synthesis of 3-alkenyl-2-oxindoles being based on expensive catalysts, non-commercially available precursors, and multistep time-consuming synthetic protocols, the development of an efficient, economical and short synthesis was inevitable and desirable. In this regard, we have now developed an efficient new protocol for the direct alkenylation of 2-oxindole by 6-aryl-4-methylthio-2*H*-pyran-2-one-3-carbonitriles **3** to deliver 3-alkenyl-2-oxindoles **8** in moderate yield. This procedure is quite efficient, noncatalytic, economical and easy in workup. Moreover, it opens a new avenue for the synthesis of 3-alkenyl-2-oxindoles. Furthermore, the synthesis of **8y** and its relative stability with respect to the other tautomers has been confirmed by single-crystal X-ray analysis and quantum chemical calculations. X-ray diffraction displayed various C−H^…^π, C−H^…^O and Ar-H^…^π intermolecular interactions. These interactions have been evaluated by quantum chemical calculations.

## Experimental

### General

The reagents and the solvents used in this study were of analytical grade and used without further purification. The melting points were determined on an electrically heated Townson Mercer melting point apparatus and are uncorrected. Commercial reagents were used without purification. ^1^H and ^13^C NMR spectra were measured on a Bruker WM-300 (300 MHz)/Jeol-400 (400 MHz) spectrometer. CDCl_3_ and DMSO-*d*_6_ were used as solvents. Chemical shifts are reported in parts per million (δ value) from Me_4_Si (δ 0 ppm for ^1^H NMR) or based on the middle peak of the solvent (CDCl_3_) (δ 77.00 ppm for ^13^C NMR) as an internal standard. Signal patterns are indicated as s, singlet; bs, broad singlet; d, doublet; dd, double doublet; t, triplet; m, multiplet; bh, broad hump. Coupling constants (*J*) are given in hertz. Infrared (IR) spectra were recorded on a Perkin-Elmer AX-1 spectrophotometer in KBr disc and are reported in wave number (cm^−1^). ESIMS spectrometers were used for mass spectra analysis.

#### Synthesis of 1-phenyl-3-(methylthio)-5*H*-dibenzo[*d,f*][1,3]diazepin-6(7*H*)-one (**5**)

A mixture of indoline-2-one (**4**, 1.1 mmol) and 2-pyranone (**3**, 1.0 mmol) and *t*-BuOK (2.1 mmol) in methanol/*t*-BuOH (10 mL) was heated under reflux for 6 h and was monitored by TLC. After completion of the reaction, the excess of solvent was removed under reduced pressure and the reaction mixture was poured onto crushed ice with vigorous stirring. The aqueous reaction mixture was neutralized with dilute HCl and the resulting precipitate was filtered, washed with water and dried. The crude product was purified by silica gel column chromatography using chloroform/hexane as eluent to afford product **5** (analytical data and spectra are given in Supporting Information File 1 and Supporting Information File 2).

#### General procedure for the synthesis of alkenylindoline-2-ones **8y**

A mixture of sodium hydride (2.1 mmol), indolin-2-one (**4**, 1.1 mmol) and lactone **3** (1.0 mmol) in dry THF (10 mL) was heated under reflux for 4–5 h. The excess of THF was removed under reduced pressure, and the reaction mixture was poured onto crushed ice with vigorous stirring. The aqueous reaction mixture was neutralized with dilute HCl. The precipitate obtained was filtered, washed with water, and dried. The isolated crude product **8y** was purified by silica gel column chromatography using hexane/chloroform as eluent to afford products **8ya**–**8yj** (the analytical data and spectra are given in Supporting Information File 1 and Supporting Information File 2).

#### General procedure for the synthesis of 1-aryl-3-(*sec*-amino)-5*H*-dibenzo[*d*,*f*][1,3]diazepin-6(7*H*)-one (**10**)

A mixture of lactone (**9**, 1.0 mmol) sodium hydride (2.1 mmol), and indolin-2-one (**4**, 1.1 mmol) in dry THF (10 mL) was heated under reflux for 4–5 h. The excess of solvent was removed under reduced pressure, and the reaction mixture was poured into ice-cold water under vigorous stirring. The aqueous mixture was neutralized with dilute HCl, and the precipitate obtained was filtered, washed with water, and dried. The isolated product was purified by silica gel column chromatography using hexane/chloroform as eluent to afford products **10a** and **10b** (the analytical data and spectra are given in Supporting Information File 1 and Supporting Information File 2).

#### Structure determination

Intensity data for the yellow colored crystals of **8y** and **5** were collected at 298(2) K on an OXFORD CrysAlis diffractometer system equipped with a graphite-monochromated Mo Kα radiation source, λ = 0.71073 Å. The final unit cell determination, scaling of the data, and corrections for Lorentz and polarization effects were performed with CrysAlis RED [44]. The structures were solved by direct methods (SHELXS-97) [45] and refined by a full-matrix least-squares procedure based on F^2^ [46]. All the calculations were carried out using WinGX system Ver-1.64 [47]. All nonhydrogen atoms were refined anisotropically; hydrogen atoms were located at calculated positions and refined using a riding model with isotropic thermal parameters fixed at 1.2 times the *U*_eq_ value of the appropriate carrier atom.

#### Crystal data for compound **5**

C_42_H_38_N_4_O_3_S_3_, formula mass 742.94, monoclinic space group *P*_1_2_1_/*C*_1_, *a* = 19.5001(9), *b* = 8.4923(3), *c* = 22.8927(8) Å, β = 92.088(3)°, *V* = 3788.5(3) Å^3^, *Z* = 4, *d*_calcd_ = 1.303 Mg·m^−3^, linear absorption coefficient 0.241 mm^−1^, *F*(000) = 1560, crystal size 0.29 × 0.20 × 0.18 mm, reflections collected 37984, independent reflections 9203 [R_int_ = 0.0258], Final indices [I > 2σ(I)] R_1_ = 0.0797 wR_2_ = 0.1919, R indices (all data) R_1_ = 0.1048, wR_2_ = 0.2065, gof 1.107, largest difference peak and hole 0.564 and −0.706 *e*·Å^−3^.

#### Crystal data for compound **8yc**

C_27_H_22_N_2_OS, formula mass 422.53, monoclinic space group *P*2_1_/*c*, *a* = 10.1246(14), *b* = 9.0703(11), *c* = 22.641(4) Å, β = 92.224(12)°, *V* = 2077.6(5) Å^3^, *Z* = 4, *d*_calcd_ = 1.351 Mg·m^−3^, linear absorption coefficient 0.179 mm^−1^, *F*(000) = 888, crystal size 0.30 × 0.25 × 0.18 mm, reflections collected 17655, independent reflections 4949 [R_int_ = 0.0321], Final indices [I > 2σ(I)] R_1_ = 0.0994 wR_2_ = 0.2460, R indices (all data) R_1_ = 0.1373, wR_2_ = 0.2727, gof 1.053, largest difference peak and hole 0.455 and −0.644 *e*·Å^−3^.

#### Computational details

Geometric characterization of all of the three tautomers of **8yc** was performed at the level of density functional theory (DFT) using the B3LYP functional [48–49]. For all the atoms 6-31G** basis sets were used. All calculations were performed using the Gaussian 03 program [50]. The intermolecular interaction energies were estimated at the MP2 level of theory. For the interaction energy calculations, the C–H^…^π, Ar–H^…^π and Ar–H^…^O distances were fixed for the dimer while all other degrees of freedom were relaxed in the geometry optimization. The magnitude of the energy corresponding to this dimer was subtracted from twice the energy of the monomer. The intermolecular interaction strengths are significantly weaker than either ionic or covalent bonding, therefore it was essential to perform basis set superposition error (BSSE) corrections. The BSSE corrections in the interaction energies were carried out using Boys–Bernardi scheme [51]. In this paper all interaction energies are reported after BSSE correction.

## Supporting Information

The Supporting Information features the analytical data and copies of ^13^C, ^1^H NMR, HRMS of all the compounds including crystallographic data (cif files) for compounds **5** and **8yc**. CCDC 897840 and 897838 contains the supplementary crystallographic data for compound **5** and **8yc**. These data can be obtained free of charge via http://www.ccdc.cam.ac.uk/conts/retrieving.html, or from the Cambridge Crystallographic Data Centre, 12 Union Road, Cambridge CB2 1EZ, UK; fax (+44) 1223-336-033; or email: deposit@ccdc.cam.ac.uk.

File 1 Analytical data.

File 2 HRMS, ^1^H and ^13^C NMR spectra.

File 3 Crystallographic data of **5**.

File 4 Crystallographic data of **8yc**.
