# Supplementary material for: Direct alkenylation of indolin-2-ones by 6-aryl-4-methylthio-2H-pyran-2-one-3-carbonitriles: a novel approach
Source: Beilstein J Org Chem. 2013 Apr 25;9:809–17. doi: 10.3762/bjoc.9.92 (PMC3678717; doi:10.3762/bjoc.9.92)
Supplement: File 1 — Analytical data. [file Beilstein_J_Org_Chem-09-809-s001.pdf]

Supporting Information

for

Direct alkenylation of indolin-2-ones by 6-aryl-4-methylthio-2*H*-pyran-2-one-3-carbonitriles: a novel approach

Sandeep Kumar[1], Ramendra Pratap[2], Abhinav Kumar[1], Brijesh Kumar[3], Vishnu K Tandon[1] and Vishnu Ji Ram*[1]

[1]Department of Chemistry, University of Lucknow, Lucknow-226007, India, [2]Department of Chemistry, North Campus, University of Delhi, New Delhi-110007, India and [3]Department of SAIF, Central Drug Research Institute, Lucknow-226001, India

Email: Vishnu Ji Ram - vjiram@yahoo.com

* Corresponding author

Analytical data

1-Phenyl-3-(methylthio)-5*H*-dibenzo[*d,f*][1,3]diazepin-6(7*H*)-one (5): Colorless powder; yield 137 mg, 41%; mp 235–236 °C; IR (KBr): 3246, 1695, 1560, 1360, 1219 cm^{-1}; ^{1}H NMR (400 MHz, DMSO-d_6): δ 2.49 (s, 3H), 6.45–6.55 (m, 2H), 6.90–7.16 (m, 9H), 8.70 (d, *J* = 2.2 Hz, 1H), 8.85 (d, *J* = 2.2 Hz, 1H); ^{13}C NMR (100 MHz, DMSO-d_6) δ 14.40, 116.40, 121.05, 122.52, 123.16, 124.11, 126.90, 127.59, 128.24 (2C), 128.55, 129.53 (2C), 132.54, 138.67, 140.81, 141.11, 142.12, 142.57, 164.78; HRMS *m*/*z*: $[M + H]^+$ calcd for $C_{20}H_{17}N_2OS$, 333.1061; found, 333.1058.

(2*Z*,5*E*)-5-(4-Fluorophenyl)-5-(1-methyl-2-oxoindolin-3-ylidene)-3-(methylthio)pent-2-enenitrile (8ya): Pale yellow crystals; yield 88%; mp 138–140 °C, IR (KBr): 3764, 3698, 3564, 3471, 2210, 1689, 1606, 1472, 1219, 766 cm^{-1}; ^{1}H NMR (400 MHz, $CDCl_3$) δ 2.19 (s, 3H), 3.25 (s, 3H), 4.71 (s, 1H), 4.89 (s, 2H), 6.10 (d, *J* = 7 Hz, 1H), 6.65 (t, *J* = 7 Hz, 1H), 6.75 (d, *J* = 7 Hz, 1H), 7.14–7.29 (m, 5H); ^{13}C NMR (100 MHz, $CDCl_3$) δ 14.94, 25.91, 38.83, 89.38, 107.81, 116.08, 116.30, 116.59, 121.75, 121.89, 123.23, 126.68, 129.30, 129.54, 142.69, 148.97, 161.67, 164.33, 167.46; HRMS *m*/*z*: $[M + H]^+$ calcd for $C_{21}H_{18}FN_2OS$, 365.1124; found, 365.1124.

(2*Z*,5*E*)-5-(4-Bromophenyl)-5-(1-methyl-2-oxoindolin-3-ylidene)-3-(methylthio)pent-2-enenitrile (8yb): Pale yellow crystals; yield 84%; mp 172–173 °C; ^{1}H NMR (400 MHz, $CDCl_3$) δ 2.18 (s, 3H), 3.25 (s, 3H), 4.71 (s, 1H), 4.89 (s, 2H), 6.14 (d, *J* = 7 Hz, 1H), 6.67 (t, *J* = 8 Hz, 1H), 6.75 (d, *J* = 7 Hz, 1H), 7.15–7.24 (m, 3H), 7.56 (d, *J* = 8 Hz, 2H); ^{13}C NMR (100 MHz, $CDCl_3$) δ 14.95, 25.92, 38.57, 89.47, 107.85, 116.58, 121.73, 121.83, 123.16, 123.29, 126.59, 129.32, 129.43, 132.26,

137.02, 142.72, 148.52, 164.17, 167.41; HRMS *m*/*z*: $[M + H]^+$ calcd for $C_{21}H_{18}BrN_2OS$, 425.0323; found, 425.0321.

(2*Z*,5*E*)-5-(1-Methyl-2-oxoindolin-3-ylidene)-3-(methylthio)-5-(naphthalen-2-yl)pent-2-enenitrile (8yc): Pale yellow crystals; yield 92%; mp 172–173 °C; IR (KBr): 3758, 3606, 3468, 2209, 1690, 1606, 1471, 759 cm^{-1}; ^1H NMR (400 MHz, $CDCl_3$) δ 2.15 (s, 3H), 3.28 (s, 3H), 4.64 (s, 1H), 4.98–5.10 (m, 2H), 6.12 (d, *J* = 8 Hz, 1H), 6.53 (t, *J* = 8 Hz, 1H), 6.75 (d, *J* = 8 Hz, 1H), 7.13 (t, *J* = 8 Hz, 1H), 7.43–7.53 (m, 3H), 7.83–7.93 (m, 4H); ^{13}C NMR (100 MHz, $CDCl_3$) δ 14.95, 25.94, 38.92, 89.29, 107.70, 116.19, 121.73, 121.80, 123.43, 125.61, 126.53, 126.81, 126.92, 127.84, 128.51, 128.71, 129.15, 133.22, 133.24, 135.90, 142.65, 150.17, 164.54, 167.69; HRMS *m*/*z*: $[M + H]^+$ calcd for $C_{25}H_{21}N_2OS$, 397.1375; found, 397.1374.

(2*Z*,5*E*)-3-(methylthio)-5-(2-oxoindolin-3-ylidene)-5-phenylpent-2-enenitrile (8yd): Pale yellow crystals; yield 48%; mp 192–193 °C; IR (KBr): 3174, 2962, 2205, 1689, 1613, 1561, 1220 cm^{-1}; ^1HNMR (400 MHz, DMSO-d_6) δ 2.23 (s, 3H), 4.77 (s, 2H), 5.18 (s, 1H), 5.92 (m, 1H), 6.53 (m, 1H), 6.79 (m, 1H), 7.08 (m, 1H), 7.29 (m, 2H), 7.48 (m, 3H), 10.70 (s, 1H); ^{13}CNMR (100 MHz, DMSO-d_6) δ 14.64, 39.00, 89.83, 109.54, 116.77, 120.74, 122.16, 122.77, 126.20, 127.0, 128.78, 128.98, 129.28, 138.30, 141.09, 149.50, 163.60, 168.45; HRMS *m*/*z*: $[M + H]^+$ calcd for $C_{20}H_{17}N_2OS$, 333.1062; found, 333.1062.

(2*Z*,5*E*)-5-(4-Bromophenyl)-3-(methylthio)-5-(2-oxoindolin-3-ylidene)pent-3-enenitrile (8ye): Pale yellow powder; yield 37%; mp 185–187 °C, IR (KBr): 3126, 2973, 2209, 1683, 1613, 1214 cm^{-1}; ^1H NMR (400 MHz, DMSO-d_6) δ 2.24 (s, 3H),

4.74 (s, 2H), 5.20 (s, 1H), 5.09 (d, J = 7 Hz, 1H), 6.62 (t, J = 8 Hz, 1H), 6.79 (d, J = 8 Hz, 1H), 7.11 (t, J = 8 Hz, 1H), 7.26 (d, J = 8 Hz, 2H), 7.70 (d, J = 8 Hz, 2H), 10.73 (s, 1H); HRMS *m*/*z*: $[M + H]^+$ calcd for $C_{20}H_{16}BrN_2OS$, 411.0166; found, 411.0164.

(2*Z*,5*E*)-5-(4-Chlorophenyl)-3-(methylthio)-5-(2-oxoindolin-3-ylidene)pent-3-enenitrile (8yf): Pale yellow powder; yield 42%; mp 176–177 °C; IR (KBr): 3122, 2987, 2212, 1674, 1212 cm^{-1}; ^1H NMR (400 MHz, DMSO-d_6) δ 2.23 (s, 3H), 4.75 (s, 2H), 5.20 (s, 1H), 5.93 (d, J = 8 Hz, 1H), 6.61 (t, J = 8 Hz, 1H), 6.79 (d, J = 7 Hz, 1H), 7.10 (t, J = 8 Hz, 1H), 7.32 (d, J = 8 Hz, 2H), 7.58 (d, J = 8 Hz, 2H), 10.74 (s, 1H); ^{13}C NMR (100 MHz, DMSO-d_6) δ 14.65, 38.00, 89.91, 109.67, 116.74, 120.93, 121.90, 122.72, 126.45, 129.14 (2C), 129.23 (2C), 129.52, 133.46, 137.02, 141.18, 148.01, 163.41, 168.30; HRMS *m*/*z*: $[M + H]^+$ calcd for $C_{20}H_{16}ClN_2OS$, 367.0672; found, 367.0670.

(2*Z*,5*E*)-5-(4-Fluorophenyl)-3-(methylthio)-5-(2-oxoindolin-3-ylidene)pent-3-enenitrile (8yg): Pale yellow powder; yield 35%; mp 180–181 °C; IR (KBr): 3129, 2228, 1678, 1217 cm^{-1}; ^1H NMR (400 MHz, DMSO-d_6) δ 2.23 (s, 3H), 4.75 (s, 2H), 5.19 (s, 1H), 5.89 (d, J = 8 Hz, 1H), 6.59 (t, J = 9 Hz, 1H), 6.78 (d, J = 7 Hz, 1H), 7.08 (t, J = 9 Hz, 1H), 7.33 (m, 4H), 10.7 (s, 1H); ^{13}C NMR (100 MHz, DMSO-d_6) δ 14.65, 39.00, 89.91, 109.63, 115.96, 116.18, 116.76, 120.90, 122.05, 122.73, 126.55, 129.43, 129.48, 129.57, 141.14, 148.45, 163.53, 168.36; HRMS *m*/*z*: $[M + H]^+$ calcd for $C_{20}H_{16}FN_2OS$, 351.0967; found, 351.0966.

(2*Z*,5*E*)-3-(methylthio)-5-(2-oxoindolin-3-ylidene)-5-(pyridin-4-yl)pent-2-enenitrile (8yh): Brown powder; yield 36%; mp 188–190 °C; IR (KBr): 3441, 2235,

1648, 1621 1219 cm^{-1}; ^{1}H NMR (400 MHz, $CDCl_3$) δ 2.20 (s, 3H), 473 (s, 1H), 4.87 (s, 2H), 6.08 (d, *J* = 8 Hz, 1H), 6.64 (t, *J* = 8 Hz, 1H), 6.79 (d, *J* = 7 Hz, 1H), 7.10–7.29 (m, 3H), 7.35 (bs, 2H), 8.74 (bs, 2H); HRMS *m*/*z*: [M + H]$^+$ calcd for $C_{20}H_{18}N_3OS$, 348.1171; found, 348.1171.

(2*Z*,5*E*)-5-(4-Methoxyphenyl)-3-(methylthio)-5-(2-oxoindolin-3-ylidene)pent-3-enenitrile (8yi): Pale yellow powder; yield 39%; mp 157–158 °C; IR (KBr): 3441, 2230, 1623, 1219 cm^{-1}; ^{1}H NMR (400 MHz, $CDCl_3$) δ 2.12 (s, 3H), 3.81 (s, 3H) 4.66 (s, 1H), 4.83 (s, 2H), 6.22 (d, *J* = 6 Hz, 1H), 6.58 (t, *J* = 8 Hz, 1H), 6.74 (d, *J* = 8 Hz, 1H), 6.91 (d, *J* = 9 Hz, 2H), 7.04 (t, *J* = 8 Hz, 1H), 7.20 (d, *J* = 6 Hz, 2H), 8.18 (s, 1H); ^{13}C NMR (100 MHz, $CDCl_3$) δ 14.99, 38.90, 55.28, 89.17, 109.42, 114.31, 116.81, 121.62, 123.03, 123.64, 126.35, 129.01, 129.12, 130.36, 139.69, 151.12, 160.11, 164.87, 169.38; HRMS *m*/*z*: [M + H]$^+$ calcd for $C_{21}H_{19}N_2O_2S$, 363.1167; found, 363.1165.

(2*Z*,5*E*)-3-(Methylthio)-5-(naphthalen-2-yl)-5-(2-oxoindolin-3-ylidene)pent-3-enenitrile (8yj): Pale yellow crystals; yield 28%; mp 161–162 °C; ^{1}HNMR (400 MHz, DMSO-d_6) δ 2.21 (s, 3H), 4.79 (d, *J* = 13 Hz, 1H), 5.01 (d, *J* = 13 Hz, 1H), 5.14 (s, 1H), 5.86 (d, *J* = 6 Hz, 1H), 6.44 (s, 1H), 6.80 (d, *J* = 7 Hz, 1H), 7.06 (s, 1H), 7.42 (d, *J* = 8 Hz, 1H), 7.57 (s, 1H), 7.89–8.04 (m, 5H), 10.76 (s, 1H); ^{13}C NMR (100 MHz, DMSO-d_6) δ 14.65, 38.09, 89.84, 109.60, 117.10, 120.78, 122.11, 122.74, 125.42, 125.91, 126.27, 126.63, 126.84, 127.81, 128.31, 128.55, 129.37, 132.70, 132.75, 136.11, 141.16, 149.41, 163.77, 168.53; HRMS *m*/*z*: [M + H]$^+$ calcd for $C_{24}H_{19}N_2OS$, 383.1218; found, 383.1218.

1-Phenyl-3-(piperidin-1-yl)-5*H*-dibenzo[*d*,*f*][1,3]diazepin-6(7*H*)-one (10a): Colorless powder; yield 48%; mp 272–275 °C; IR (KBr): 3452, 3240, 2931, 1687, 1222 cm^{-1}; ^{1}H NMR (400 MHz, DMSO-d_6) δ 1.58 (bs, 6H), 3.20 (bs, 4H), 6.43–6.56 (m, 3H), 6.64–6.70 (m, 4H), 6.99–7.07 (m, 3H), 7.19 (d, *J* = 6, 2H), 8.48 (s, 1H), 8.77 (s, 1H); HRMS *m*/*z*: $[M + H]^+$ calcd for $C_{24}H_{24}N_3O$, 370.1919; found, 370.1917.

1-(4-Fluorophenyl)-3-(pyrrolidin-1-yl)-5*H*-dibenzo[*d*,*f*][1,3]diazepin-6(7*H*)-one (10b): Colorless powder; yield 55%; mp 292–293 °C, IR (KBr): 3468, 2942, 1668, 1225 cm^{-1}; ^{1}H NMR (400 MHz, DMSO-d_6) δ 1.93–1.96 (m, 4H), 3.74 (d, *J* = 6 Hz, 2H), 3.90 (d, *J* = 6 Hz, 2H), 6.27–6.40 (m, 2H), 6.56–6.58 (m, 1H), 6.99–7.18 (m, 3H), 7.37 (t, *J* = 9 Hz, 2H), 8.02 (t, *J* = 8 Hz, 2H), 8.48 (s, 1H), 8.73 (s, 1H); HRMS *m*/*z*: $[M + H]^+$ calcd for $C_{23}H_9FN_3O$, 374.1669; found, 374.1668.
