# Supplementary material for: Direct alkenylation of indolin-2-ones by 6-aryl-4-methylthio-2H-pyran-2-one-3-carbonitriles: a novel approach
Source: Beilstein J Org Chem. 2013 Apr 25;9:809–17. doi: 10.3762/bjoc.9.92 (PMC3678717; doi:10.3762/bjoc.9.92)
Supplement: File 2 — HRMS, 1H and 13C NMR spectra. [file Beilstein_J_Org_Chem-09-809-s002.pdf]

Supporting Information

for

Direct alkenylation of indolin-2-ones by 6-aryl-4-methylthio-2*H*-pyran-2-one-3-carbonitriles: a novel approach

Sandeep Kumar[1], Ramendra Pratap[2], Abhinav Kumar[1], Brijesh Kumar[3], Vishnu K Tandon[1] and Vishnu Ji Ram*[1]

[1]Department of Chemistry, University of Lucknow, Lucknow-226007, India, [2]Department of Chemistry, North Campus, University of Delhi, New Delhi-110007, India and [3]Department of SAIF, Central Drug Research Institute, Lucknow-226001, India

Email: Vishnu Ji Ram - vjiram@yahoo.com

* Corresponding author

HRMS, ^{1}H and ^{13}C NMR spectra

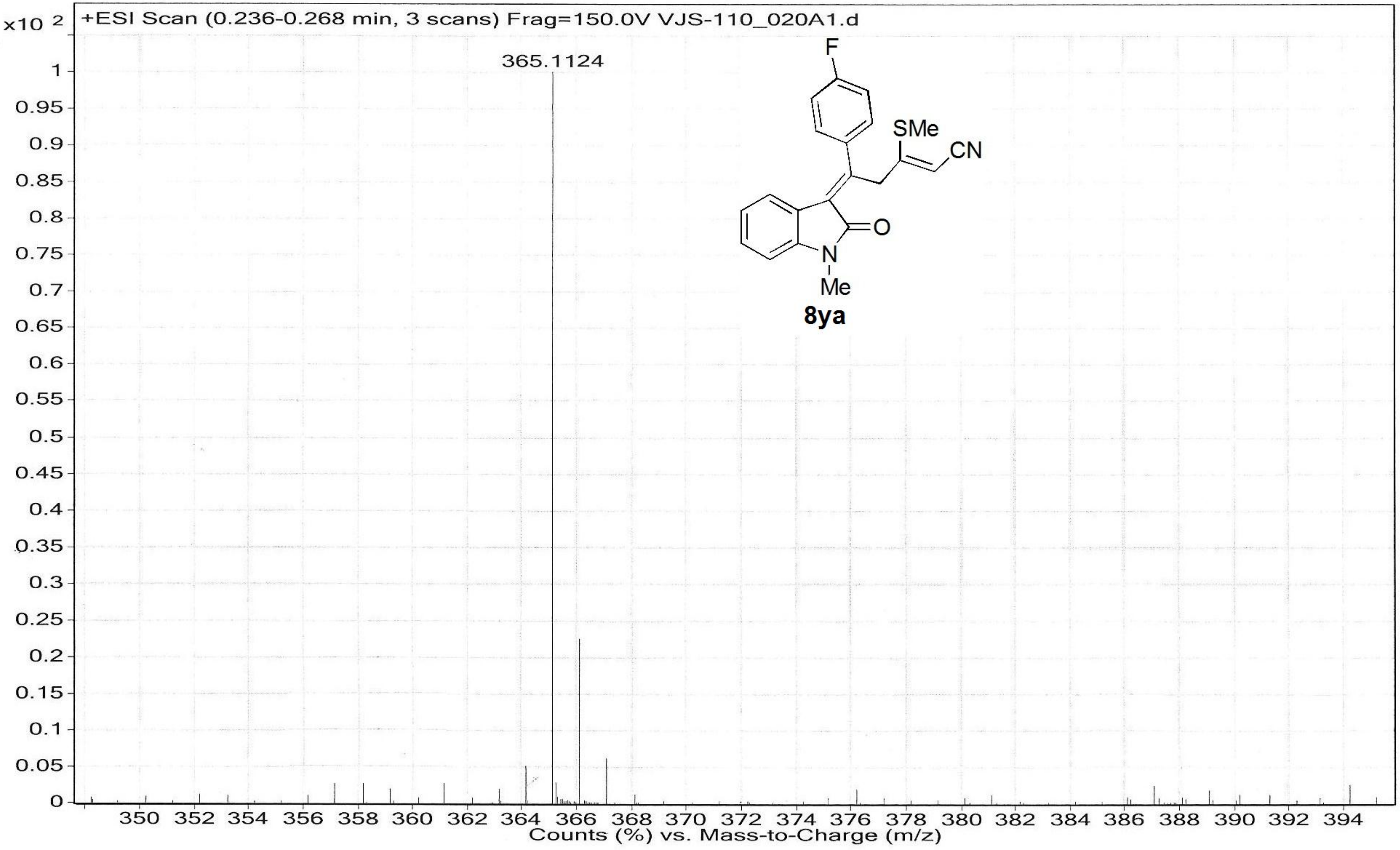
+ESI Scan (0.236-0.268 min, 3 scans) Frag=150.0V VJS-110_020A1.d
x10 2
365.1124
F
SMe
CN
O
N
Me
8ya
Counts (%) vs. Mass-to-Charge (m/z)

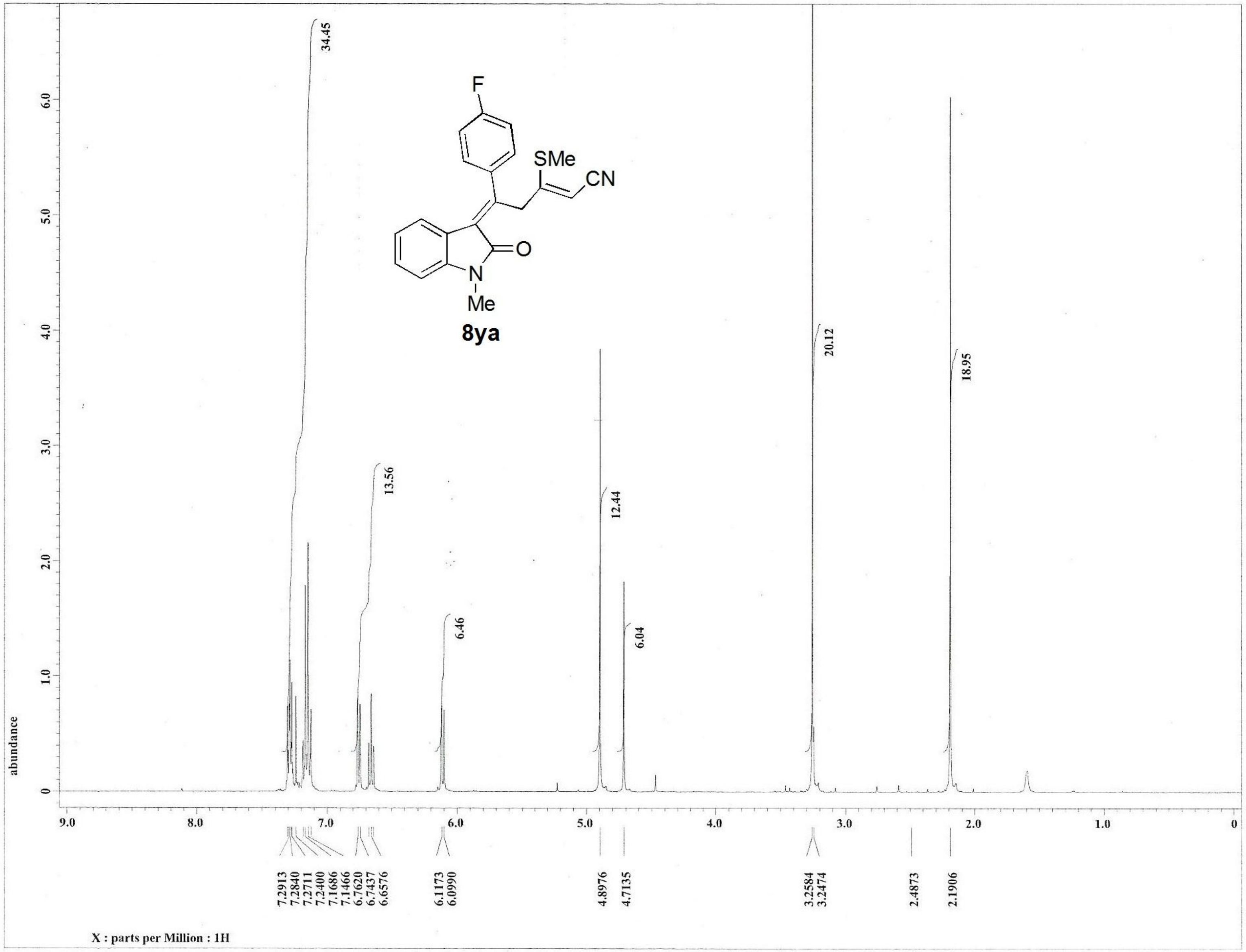
F
SMe
CN
O
N
Me
8ya
abundance
X : parts per Million : 1H
34.45
13.56
6.46
12.44
6.04
20.12
18.95
7.2913
7.2840
7.2711
7.2400
7.1686
7.1466
6.7620
6.7437
6.6576
6.1173
6.0990
4.8976
4.7135
3.2584
3.2474
2.4873
2.1906

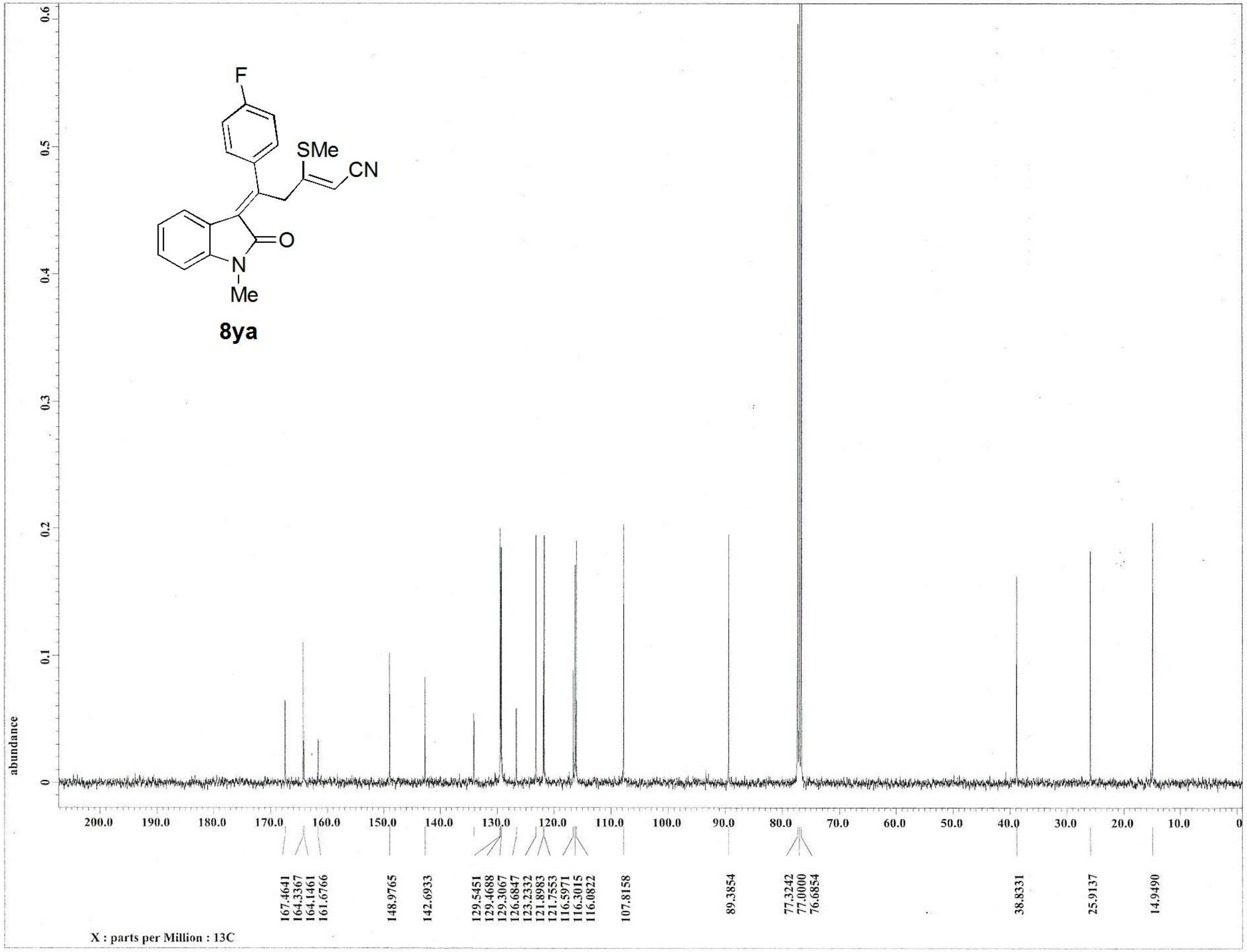
F
SMe
CN
O
N
Me
8ya
abundance
X : parts per Million : 13C
167.4641
164.3367
164.1461
161.6766
148.9765
142.6933
129.5451
129.4688
129.3067
126.6847
123.2332
121.8983
121.7553
116.5971
116.3015
116.0822
107.8158
89.3854
77.3242
77.0000
76.6854
38.8331
25.9137
14.9490

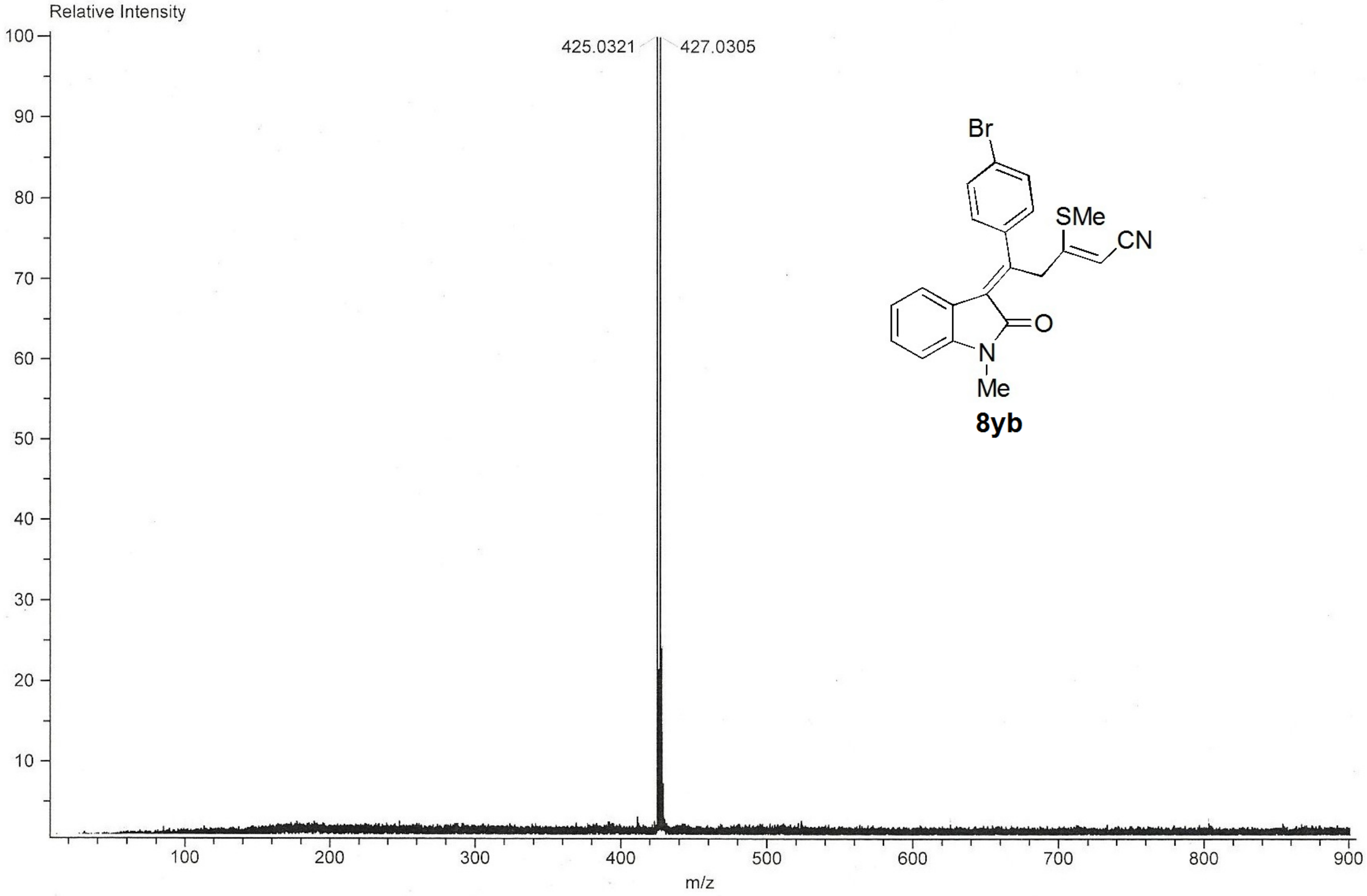
Relative Intensity
425.0321
427.0305
m/z
Br
SMe
CN
O
N
Me
8yb

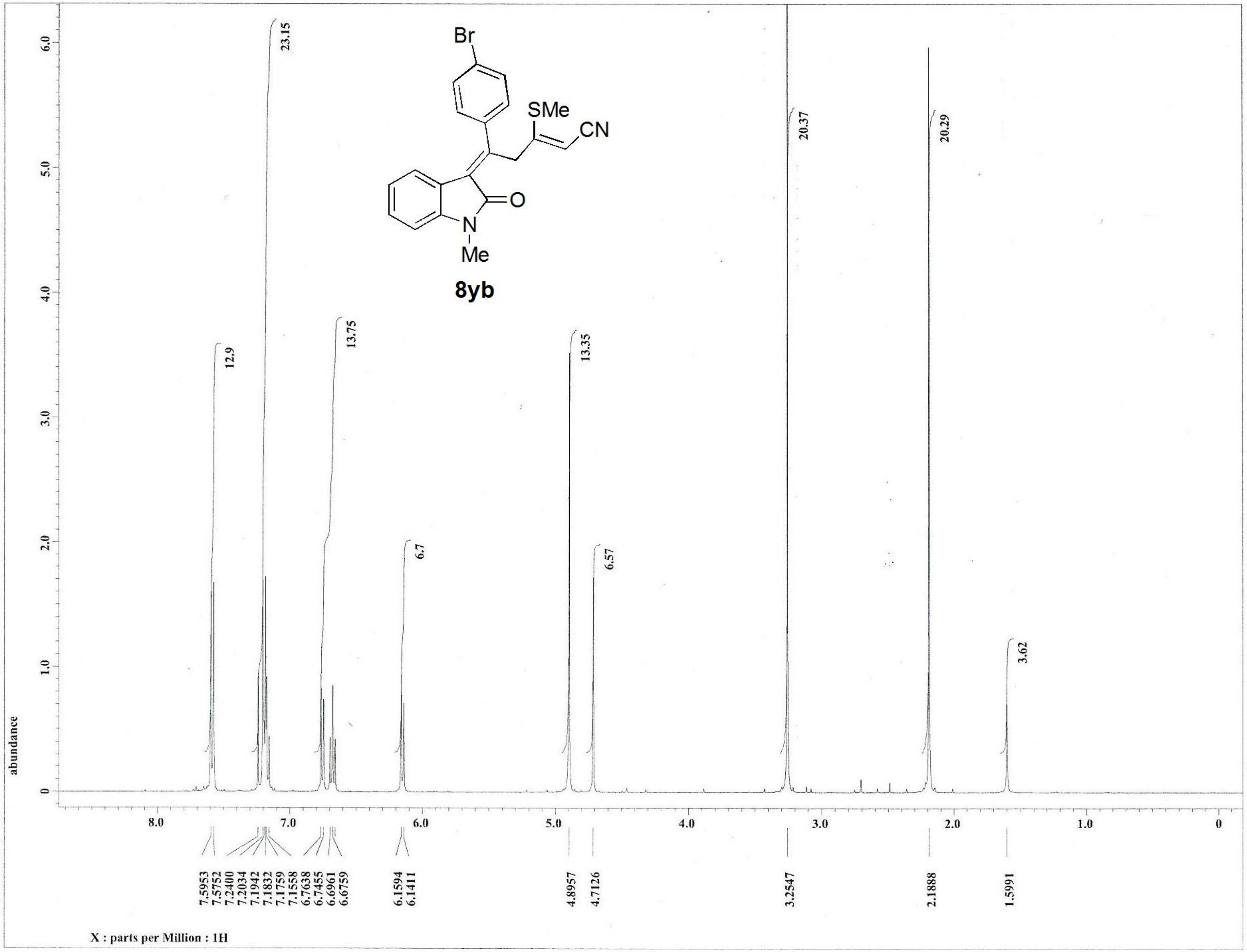
Br
SMe
CN
O
N
Me
8yb
23.15
12.9
13.75
6.7
13.35
6.57
20.37
20.29
3.62
abundance
0
1.0
2.0
3.0
4.0
5.0
6.0
8.0
7.0
6.0
5.0
4.0
3.0
2.0
1.0
0
7.5953
7.5752
7.2400
7.2034
7.1942
7.1832
7.1759
7.1558
6.7638
6.7455
6.6961
6.6759
6.1594
6.1411
4.8957
4.7126
3.2547
2.1888
1.5991
X : parts per Million : 1H

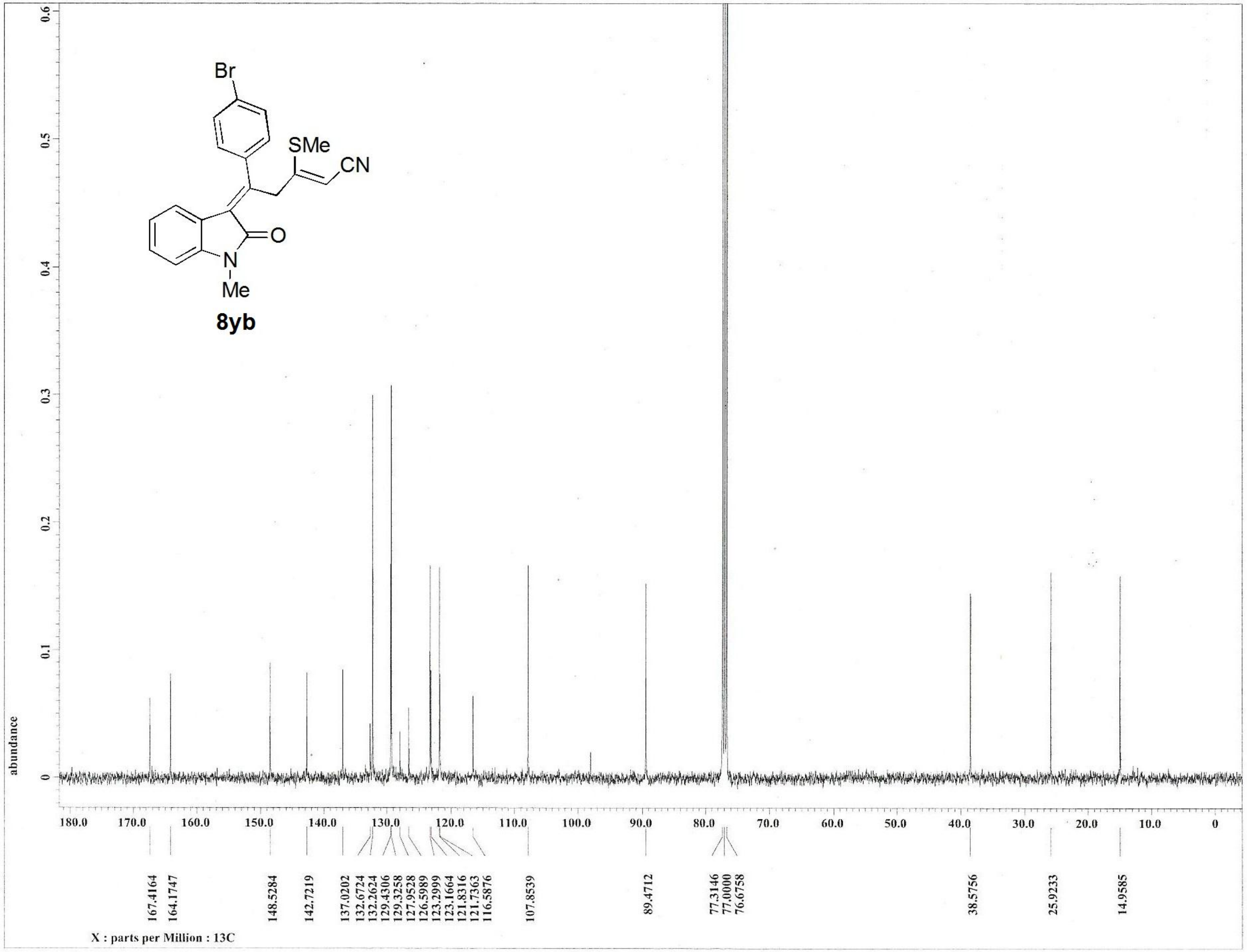

Br
SMe
CN
N
Me
O
8yb
abundance
X : parts per Million : 13C
167.4164
164.1747
148.5284
142.7219
137.0202
132.6724
132.2624
129.4306
129.3258
127.9528
126.5989
123.2999
123.1664
121.8316
121.7363
116.5876
107.8539
89.4712
77.3146
77.0000
76.6758
38.5756
25.9233
14.9585

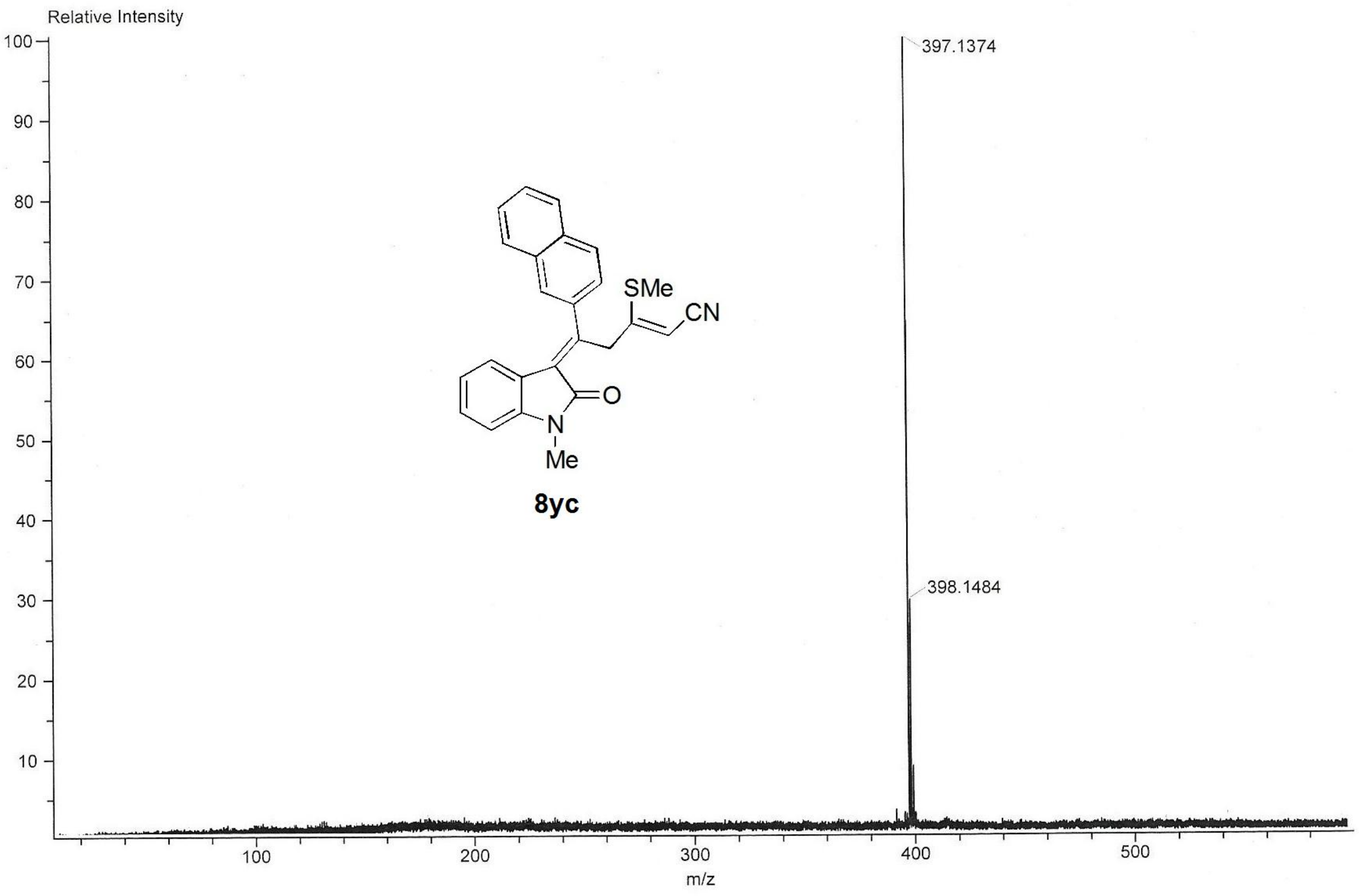
Relative Intensity
397.1374
398.1484
SMe
CN
O
N
Me
8yc
m/z

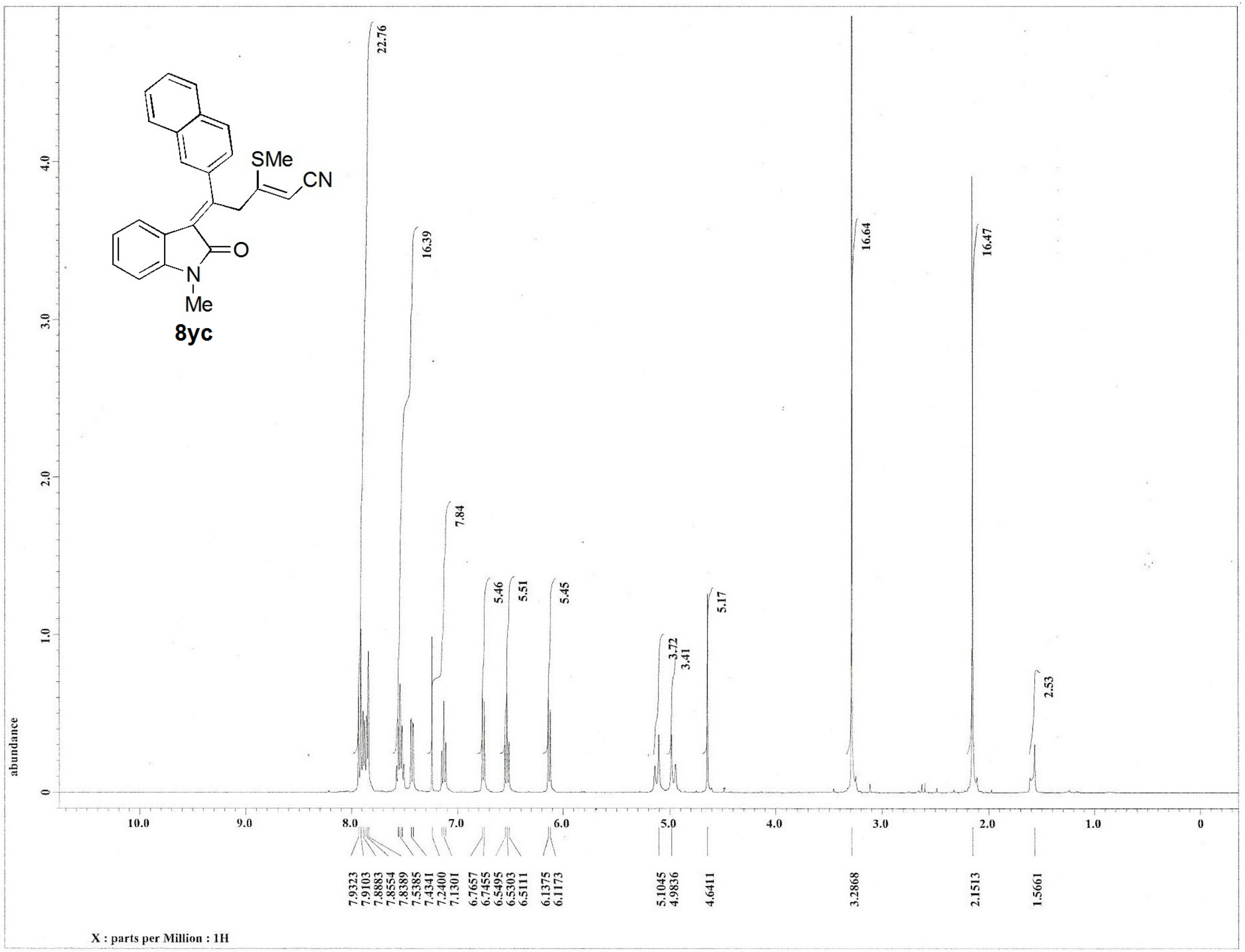
SMe
CN
O
N
Me
8yc
abundance
X : parts per Million : 1H

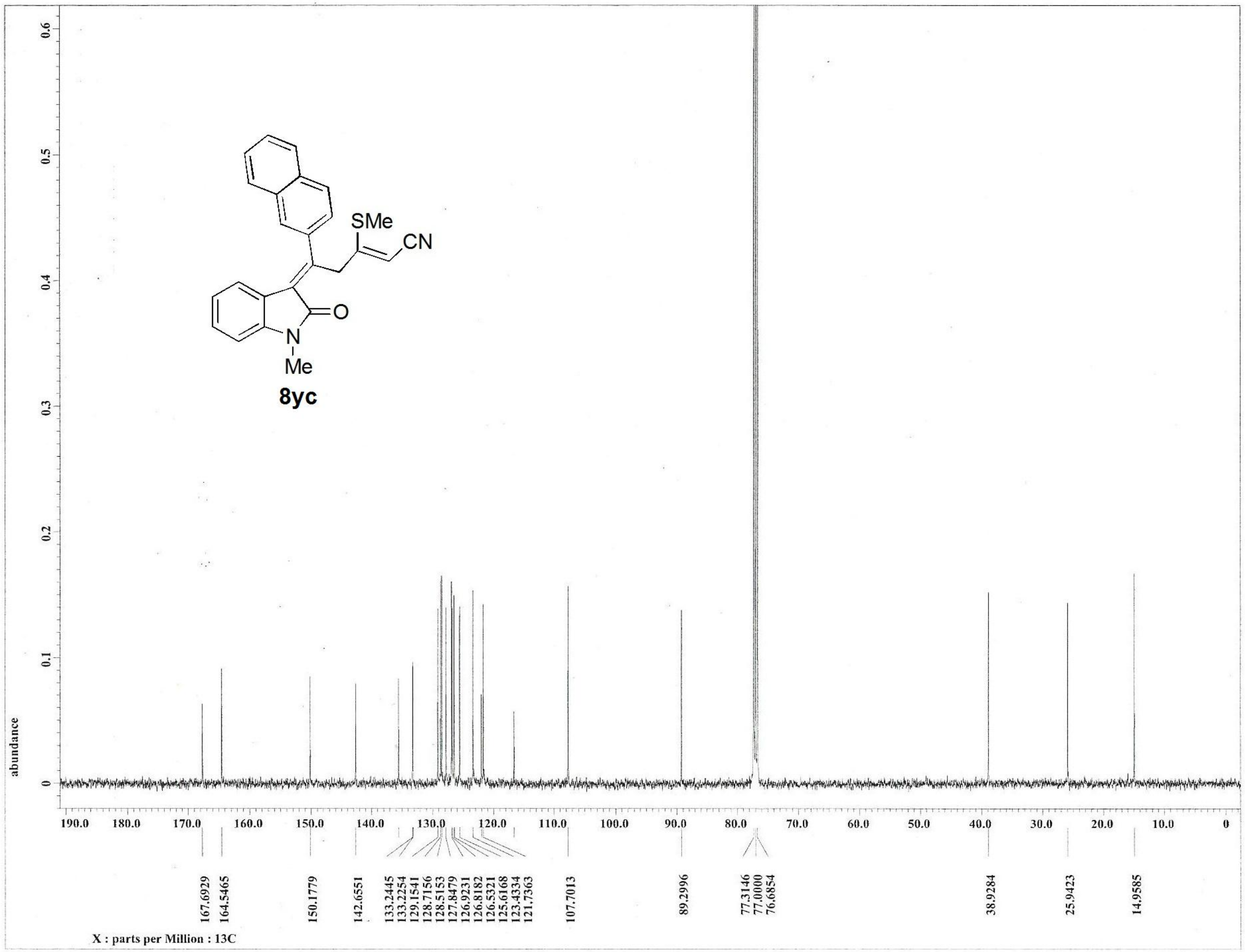
SMe
CN
N
O
Me
8yc
abundance
0.6
0.5
0.4
0.3
0.2
0.1
0
190.0 180.0 170.0 160.0 150.0 140.0 130.0 120.0 110.0 100.0 90.0 80.0 70.0 60.0 50.0 40.0 30.0 20.0 10.0 0
167.6929
164.5465
150.1779
142.6551
133.2445
133.2254
129.1541
128.7156
128.5153
127.8479
126.9231
126.8182
126.5321
125.6168
123.4334
121.7363
107.7013
89.2996
77.3146
77.0000
76.6854
38.9284
25.9423
14.9585
X : parts per Million : 13C

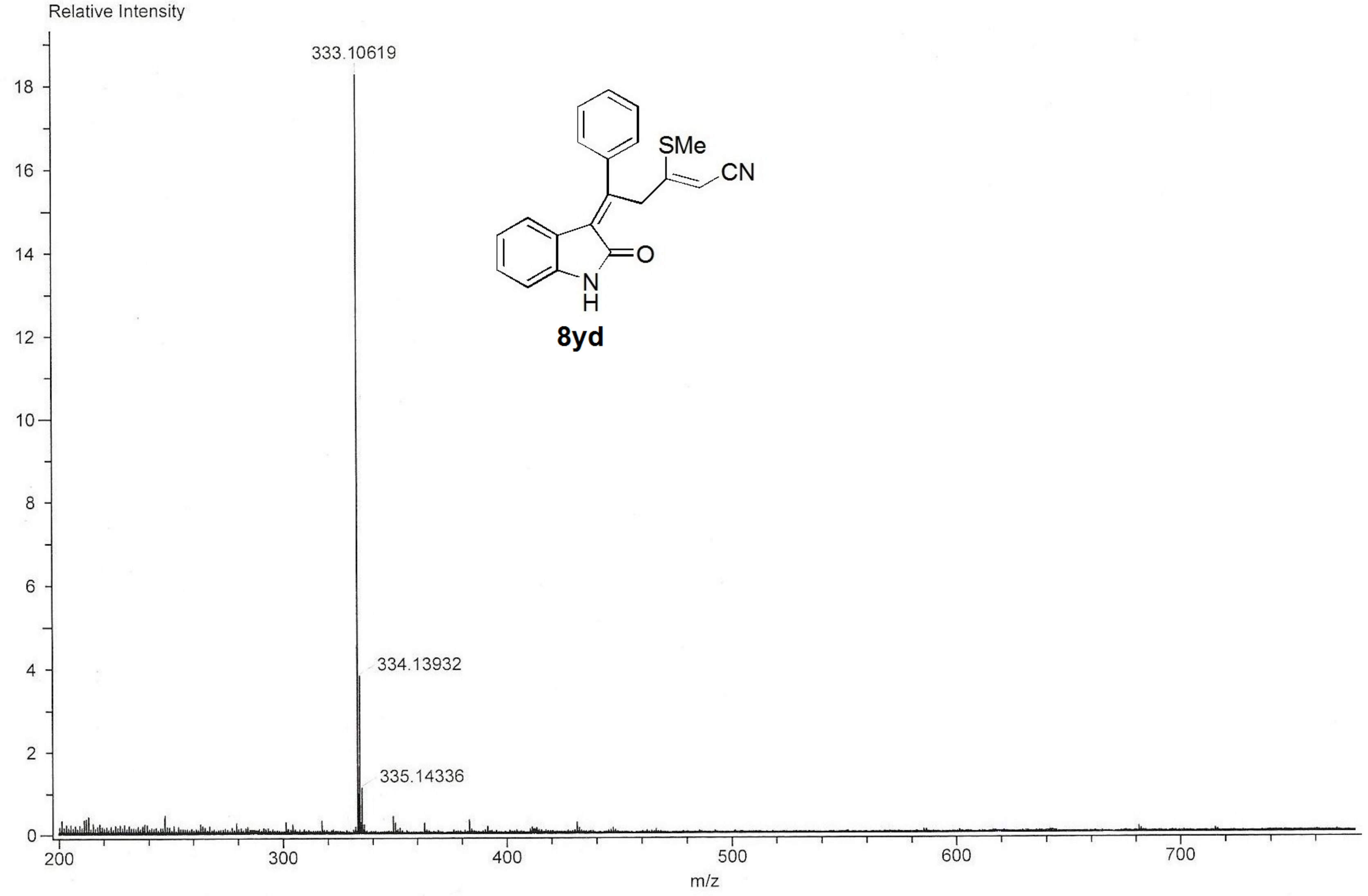
Relative Intensity
333.10619
334.13932
335.14336
SMe
CN
O
N
H
8yd
18
16
14
12
10
8
6
4
2
0
200
300
400
500
600
700
m/z

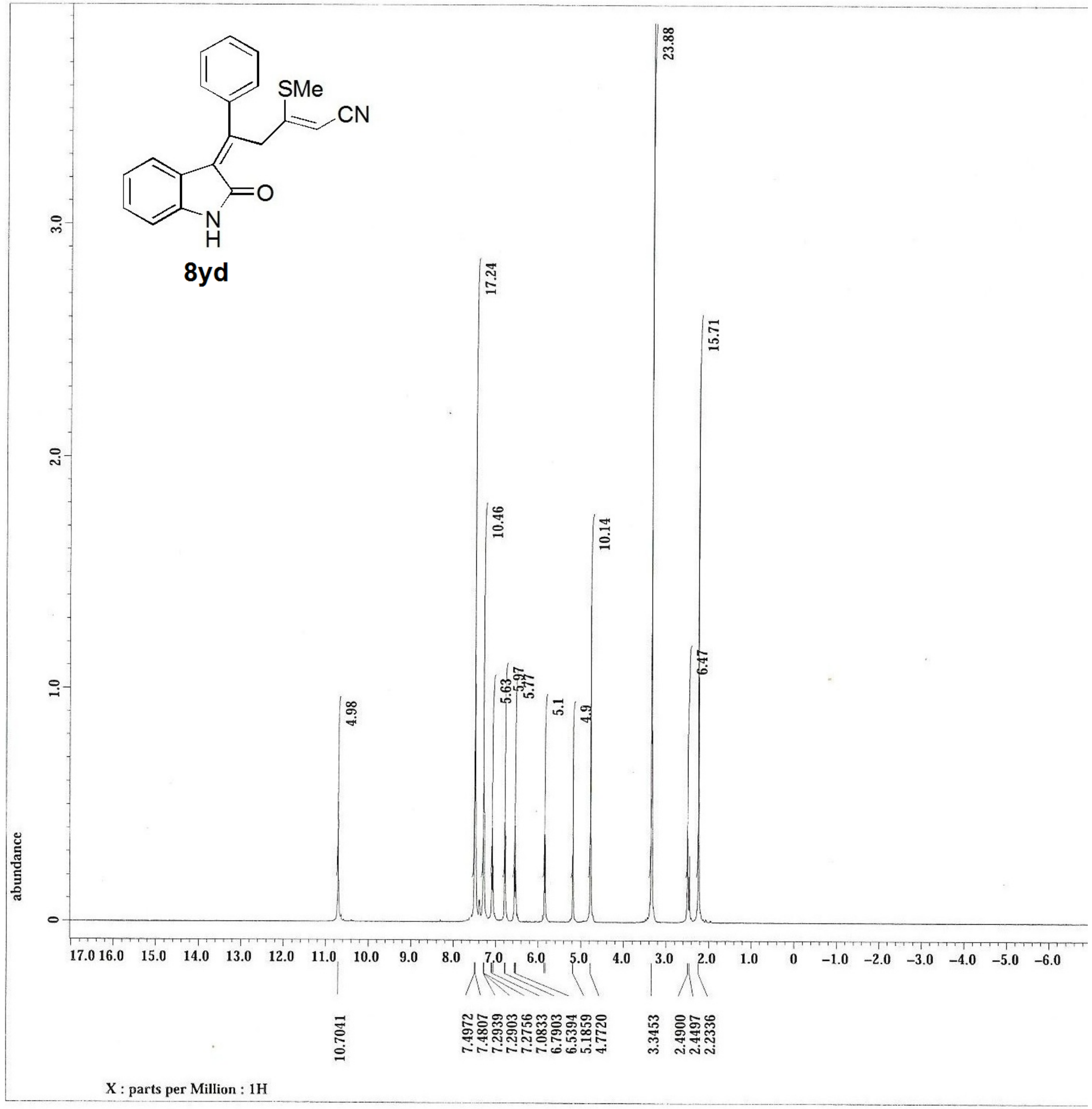
SMe
CN
N
H
O
8yd
abundance
X : parts per Million : 1H
4.98
17.24
10.46
5.63
5.97
5.77
5.1
4.9
10.14
23.88
6.47
15.71
10.7041
7.4972
7.4807
7.2939
7.2903
7.2756
7.0833
6.7903
6.5394
5.1859
4.7720
3.3453
2.4900
2.4497
2.2336

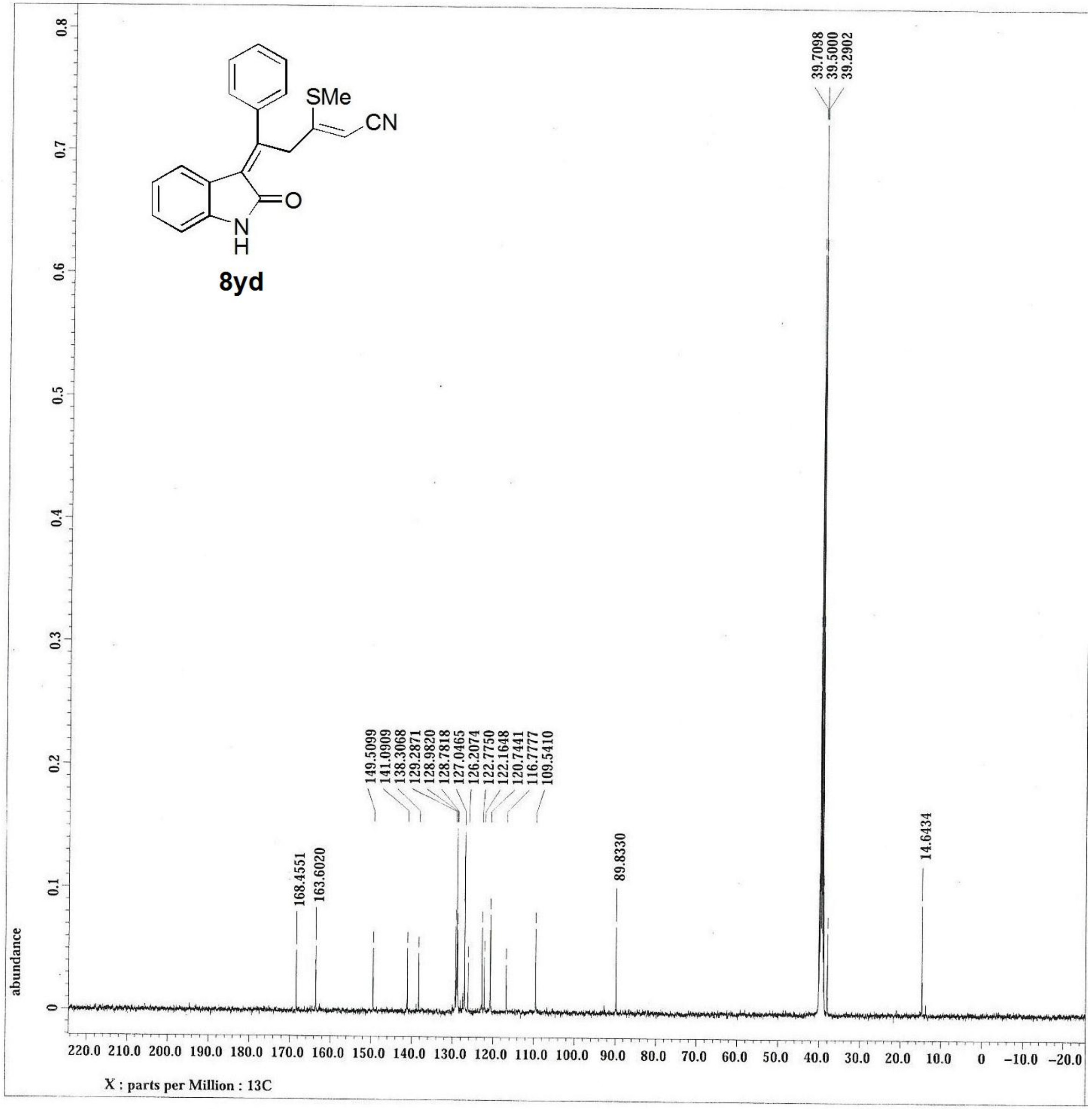
SMe
CN
N
H
O
8yd
39.7098
39.5000
39.2902
149.5099
141.0909
138.3068
129.2871
128.9820
128.7818
127.0465
126.2074
122.7750
122.1648
120.7441
116.7777
109.5410
168.4551
163.6020
89.8330
14.6434
abundance
0.8
0.7
0.6
0.5
0.4
0.3
0.2
0.1
0
220.0 210.0 200.0 190.0 180.0 170.0 160.0 150.0 140.0 130.0 120.0 110.0 100.0 90.0 80.0 70.0 60.0 50.0 40.0 30.0 20.0 10.0 0 −10.0 −20.0
X : parts per Million : 13C

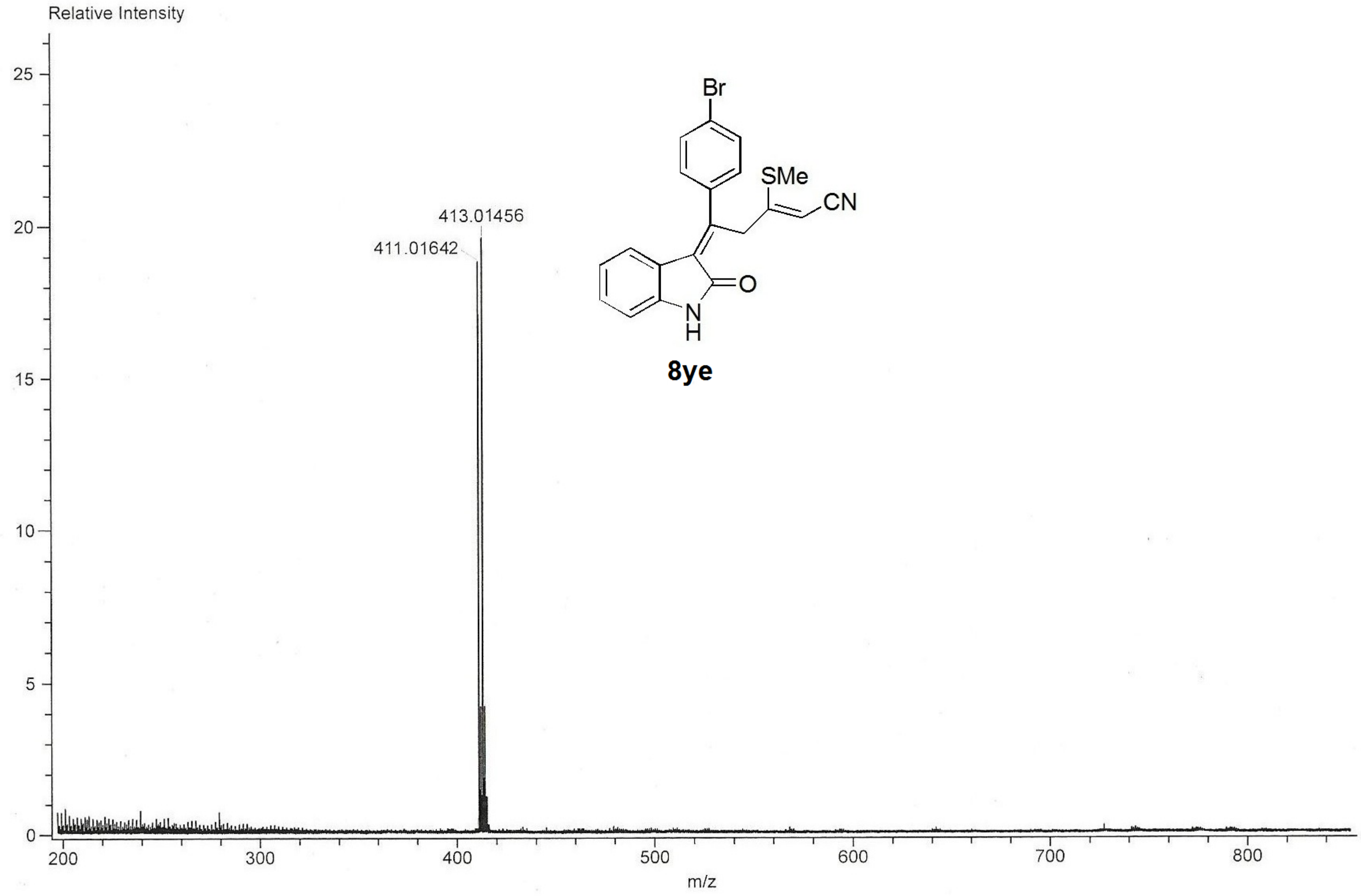

Relative Intensity
25
20
15
10
5
0
413.01456
411.01642
Br
SMe
CN
O
N
H
200
300
400
500
600
700
800
m/z

8ye

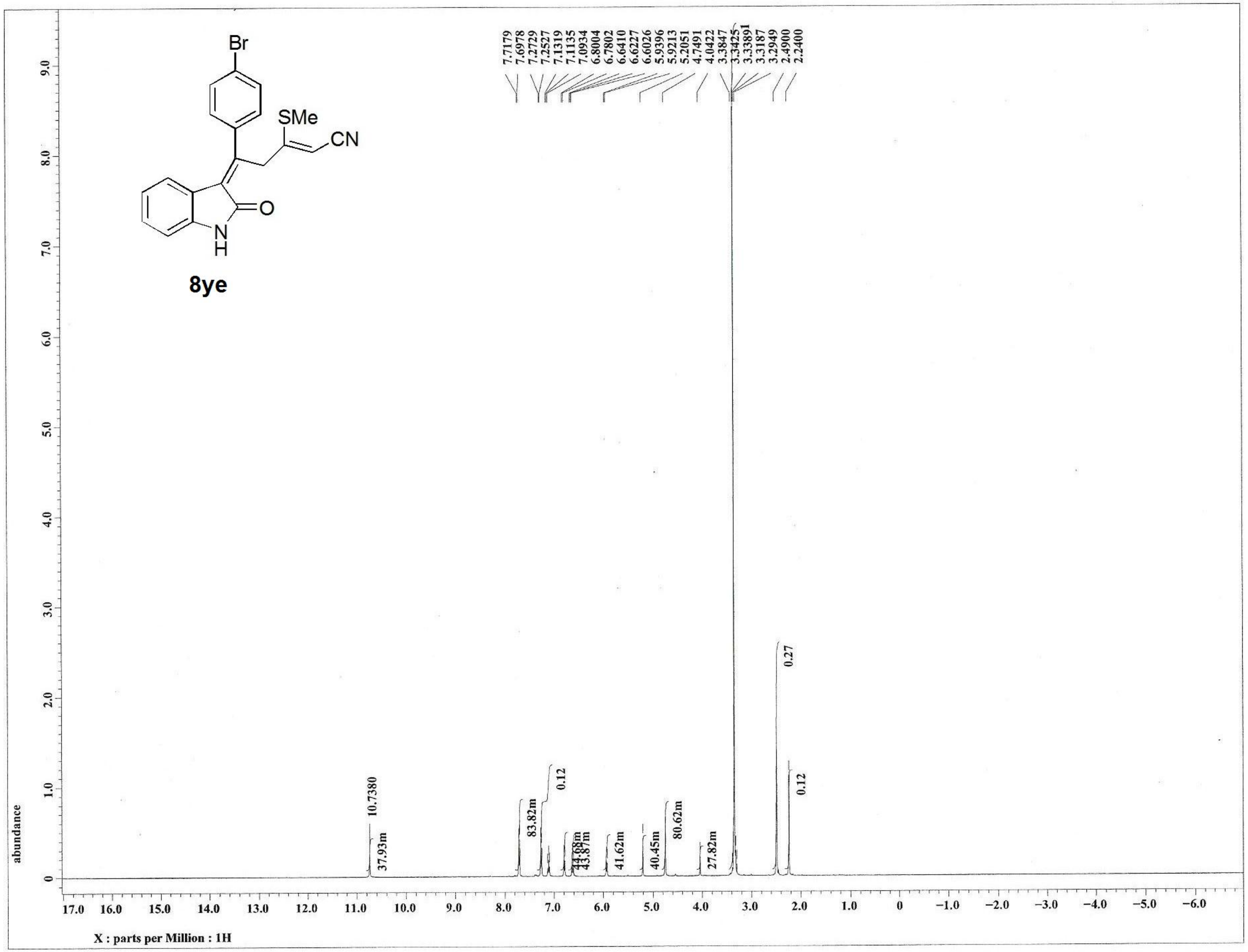
Br
SMe
CN
O
N
H
8ye
7.7179
7.6978
7.2729
7.2527
7.1319
7.1135
7.0934
6.8004
6.7802
6.6410
6.6227
6.6026
5.9396
5.9213
5.2051
4.7491
4.0422
3.3847
3.3425
3.33891
3.3187
3.2949
2.4900
2.2400
10.7380
37.93m
83.82m
0.12
43.87m
41.62m
40.45m
80.62m
27.82m
0.27
0.12
abundance
X : parts per Million : 1H

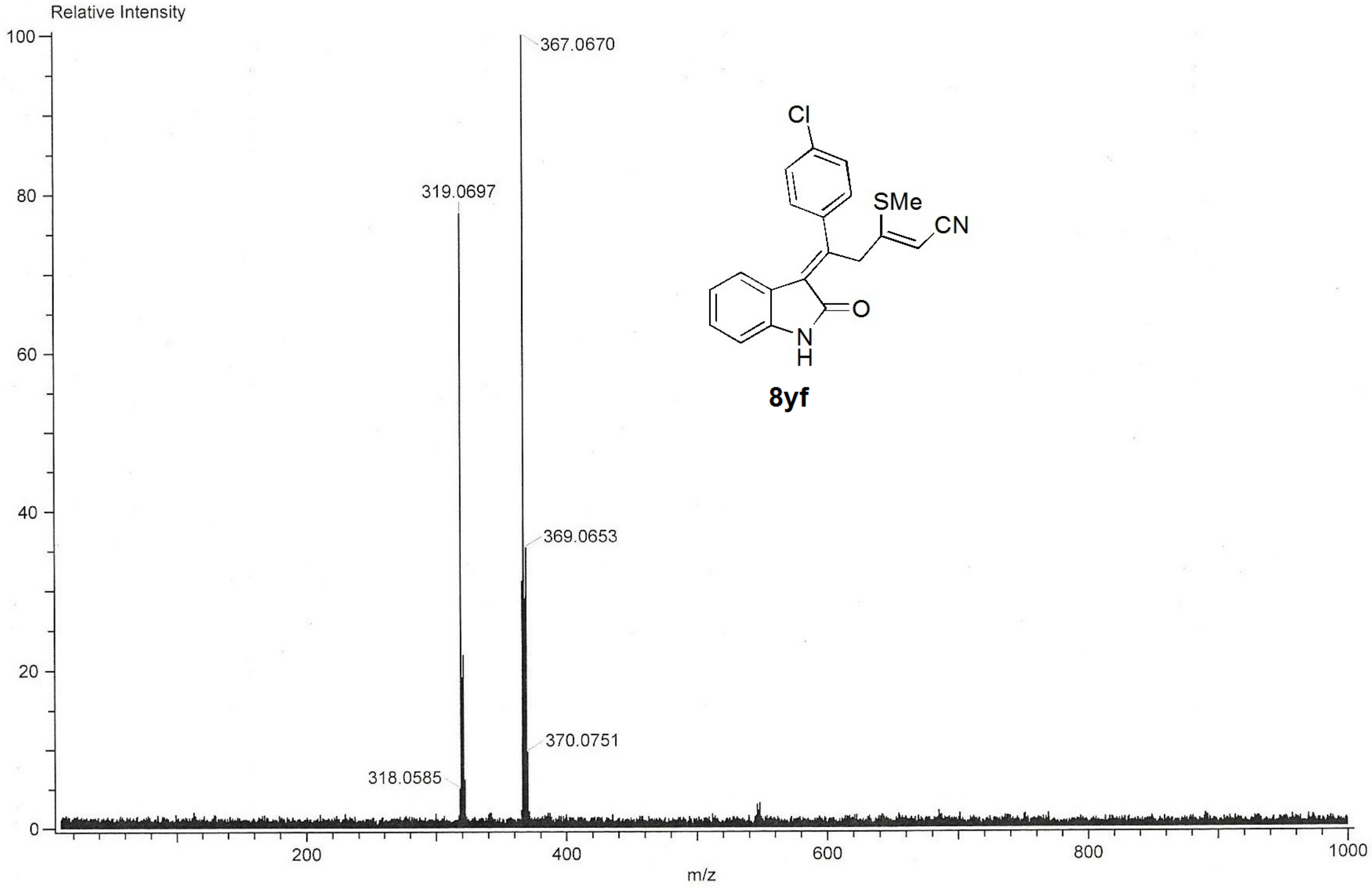
Relative Intensity
100
80
60
40
20
0
367.0670
319.0697
369.0653
370.0751
318.0585
200
400
600
800
1000
m/z
Cl
SMe
CN
N
H
O
8yf

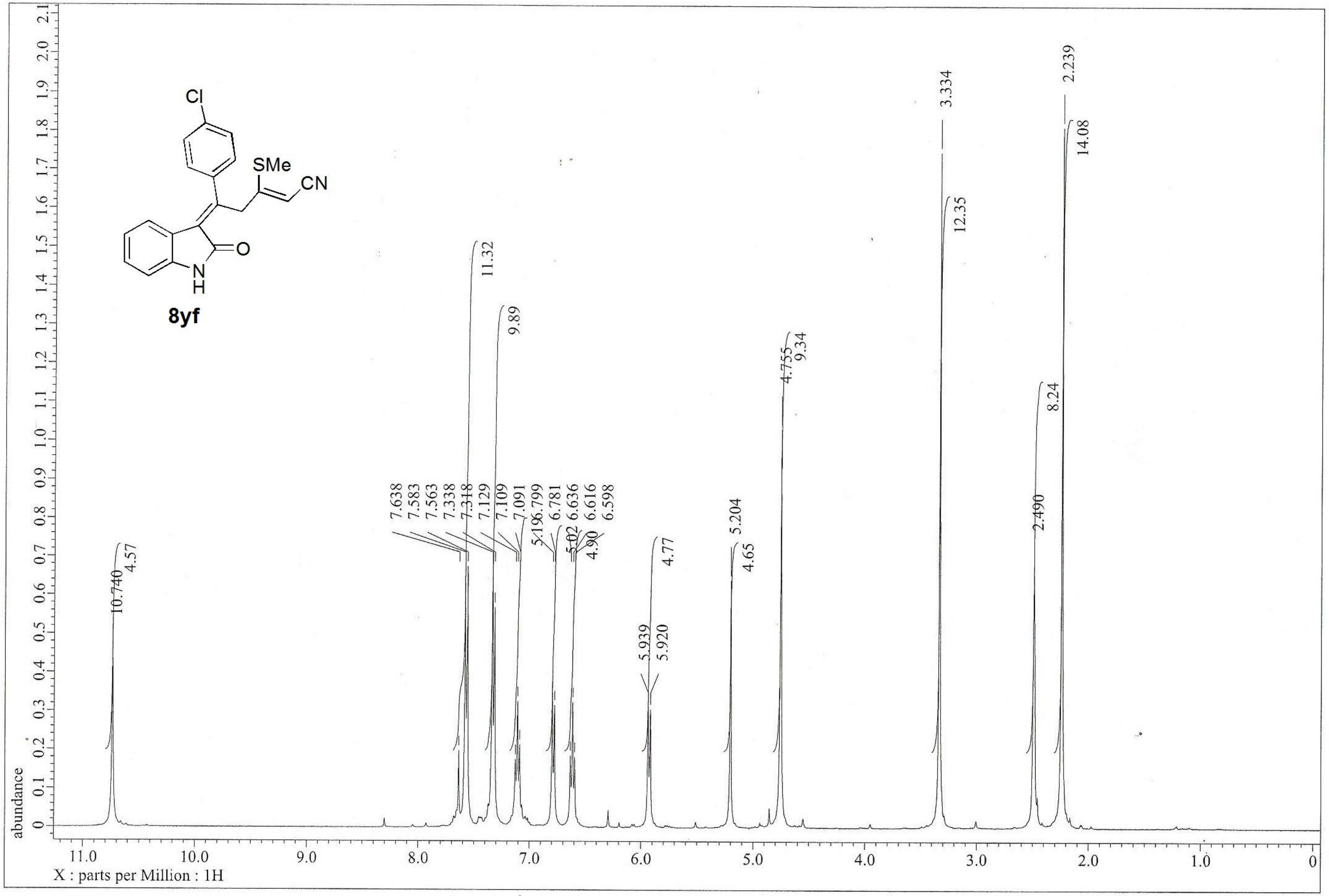

Cl
SMe
CN
N
H
O
8yf
10.740
4.57
7.638
7.583
7.563
7.338
7.318
7.129
7.109
7.091
6.799
6.781
6.636
6.616
6.598
11.32
9.89
5.939
5.920
4.77
5.204
4.65
4.755
9.34
3.334
12.35
2.490
8.24
2.239
14.08
abundance
X : parts per Million : 1H

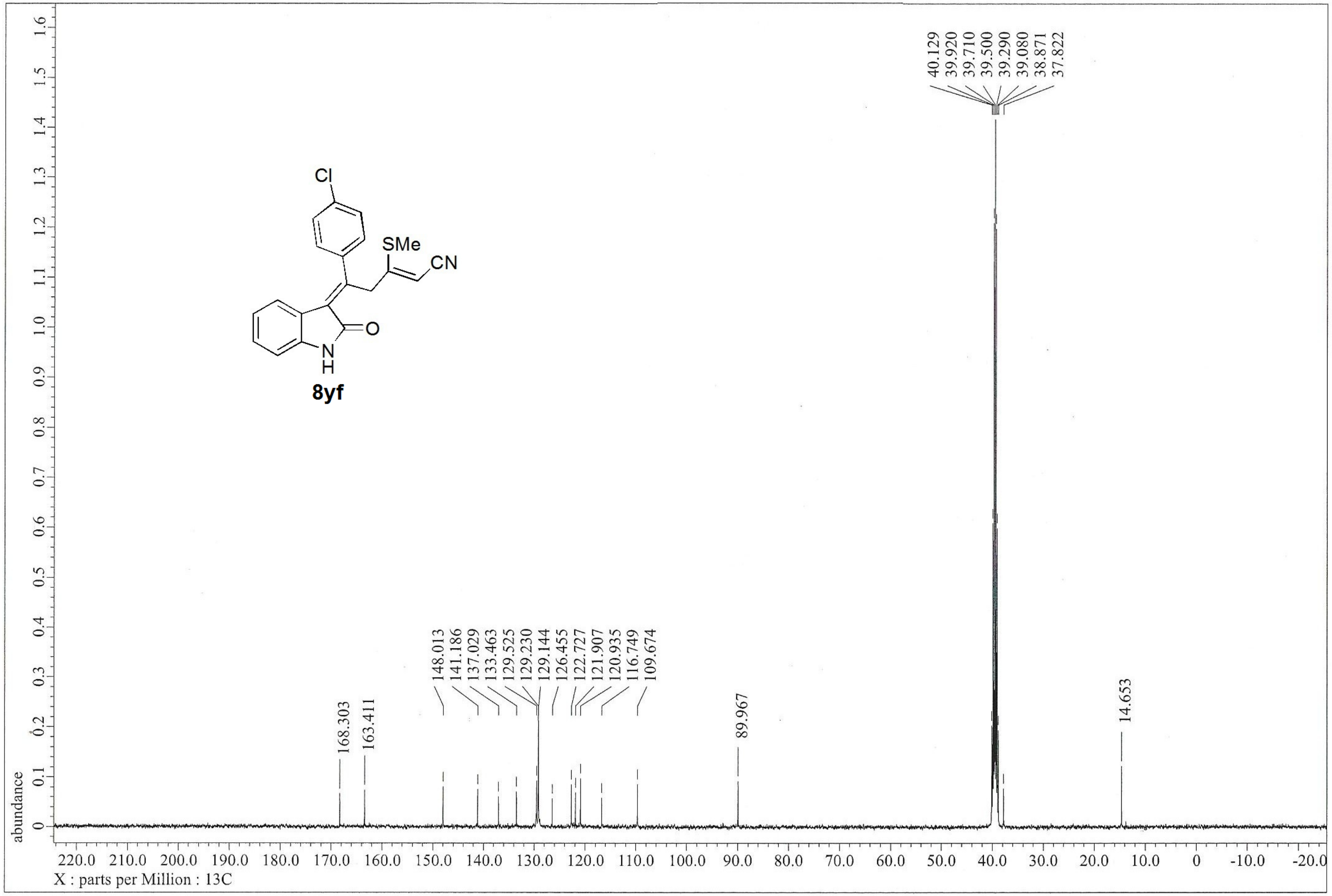

Cl
SMe
CN
O
N
H
8yf
40.129
39.920
39.710
39.500
39.290
39.080
38.871
37.822
148.013
141.186
137.029
133.463
129.525
129.230
129.144
126.455
122.727
121.907
120.935
116.749
109.674
168.303
163.411
89.967
14.653
abundance
X : parts per Million : 13C

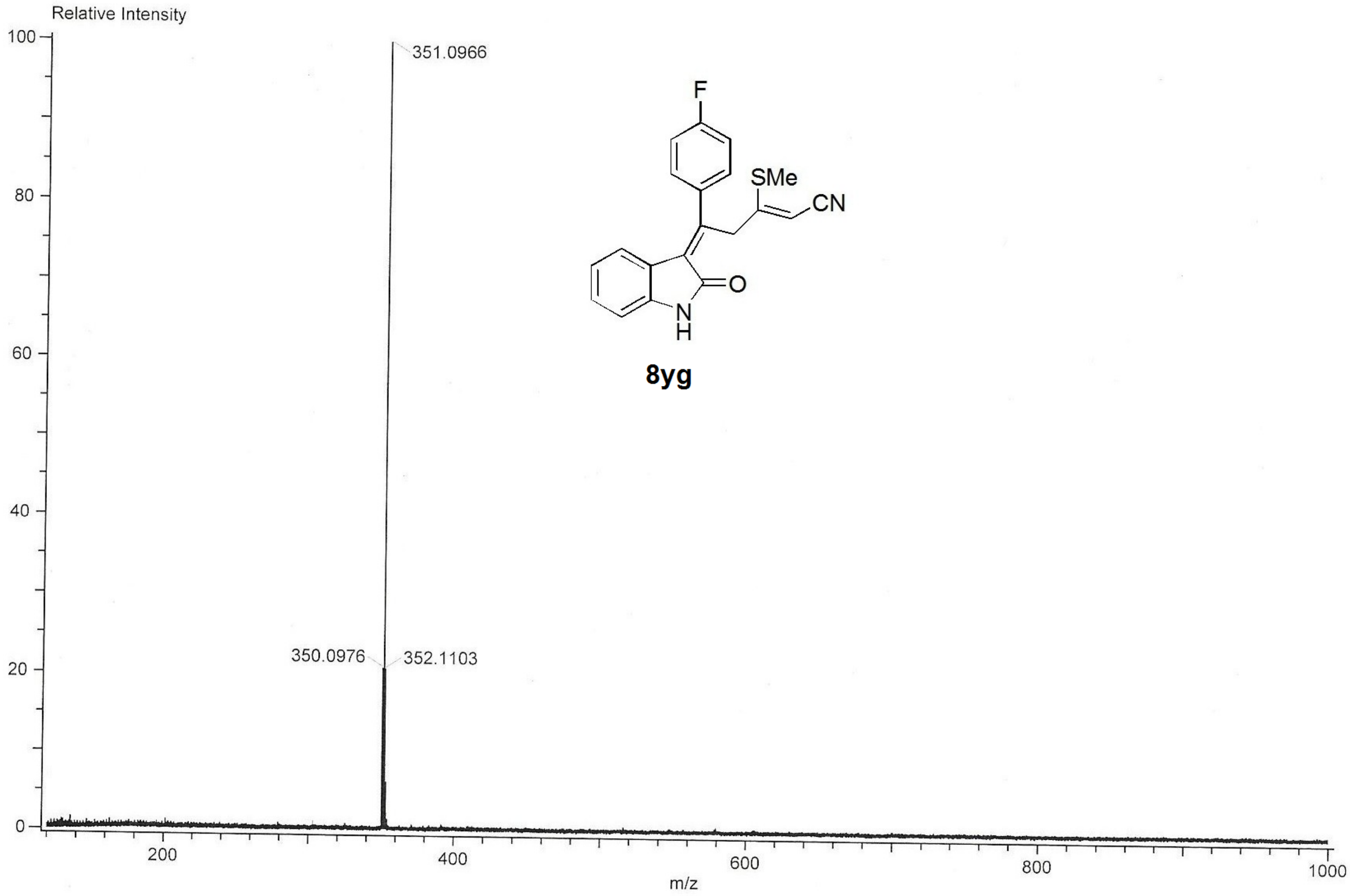
Relative Intensity
100
80
60
40
20
0
351.0966
350.0976
352.1103
F
SMe
CN
O
N
H
8yg
200
400
600
800
1000
m/z

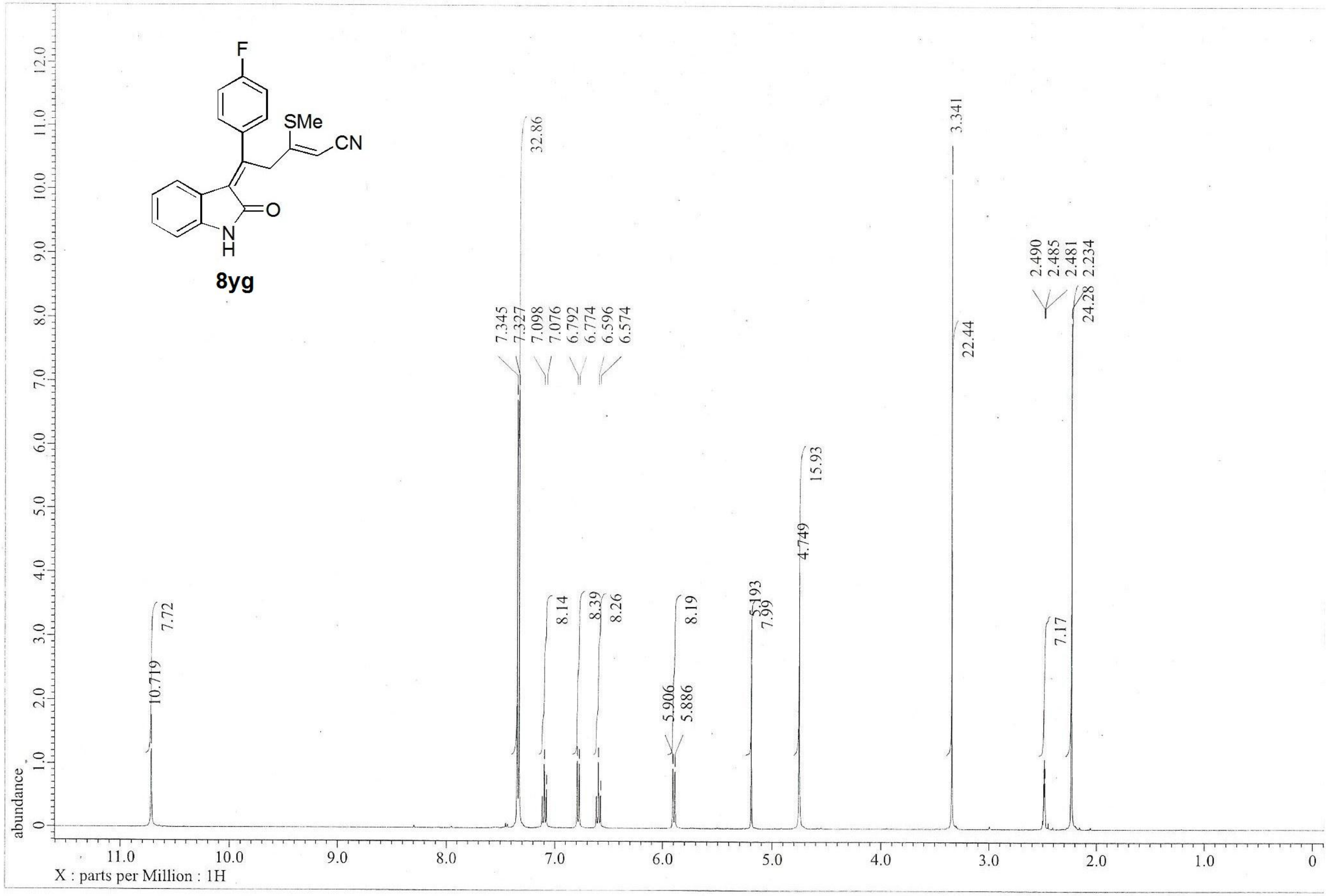

8yg
SMe
CN
F
O
N
H
10.719
7.72
7.345
7.327
7.098
7.076
6.792
6.774
6.596
6.574
32.86
8.14
8.39
8.26
5.906
5.886
8.19
5.193
7.99
4.749
15.93
3.341
22.44
2.490
2.485
2.481
2.234
7.17
24.28
abundance
X : parts per Million : 1H

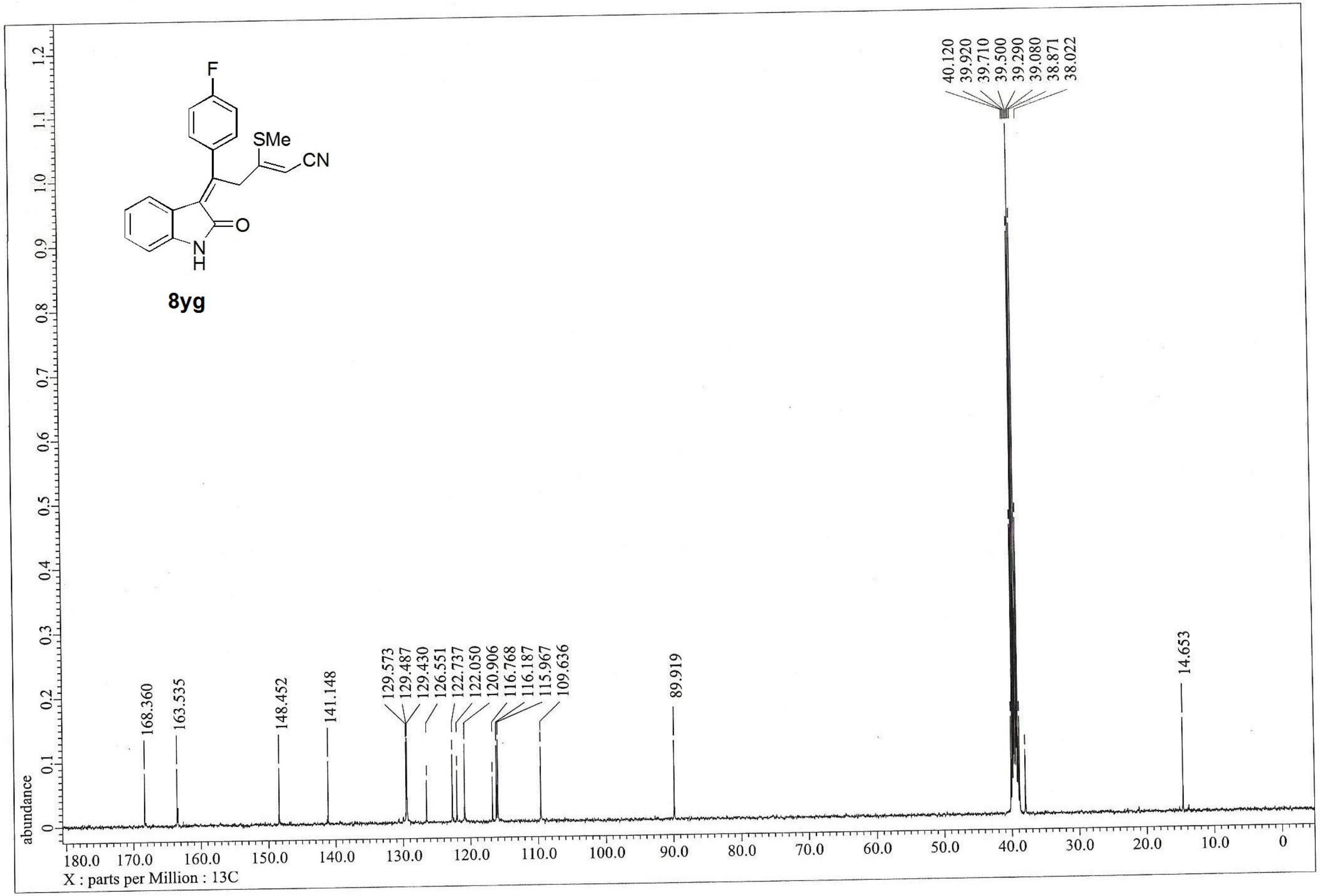
F
SMe
CN
O
N
H
8yg
40.120
39.920
39.710
39.500
39.290
39.080
38.871
38.022
168.360
163.535
148.452
141.148
129.573
129.487
129.430
126.551
122.737
122.050
120.906
116.768
116.187
115.967
109.636
89.919
14.653
abundance
X : parts per Million : 13C

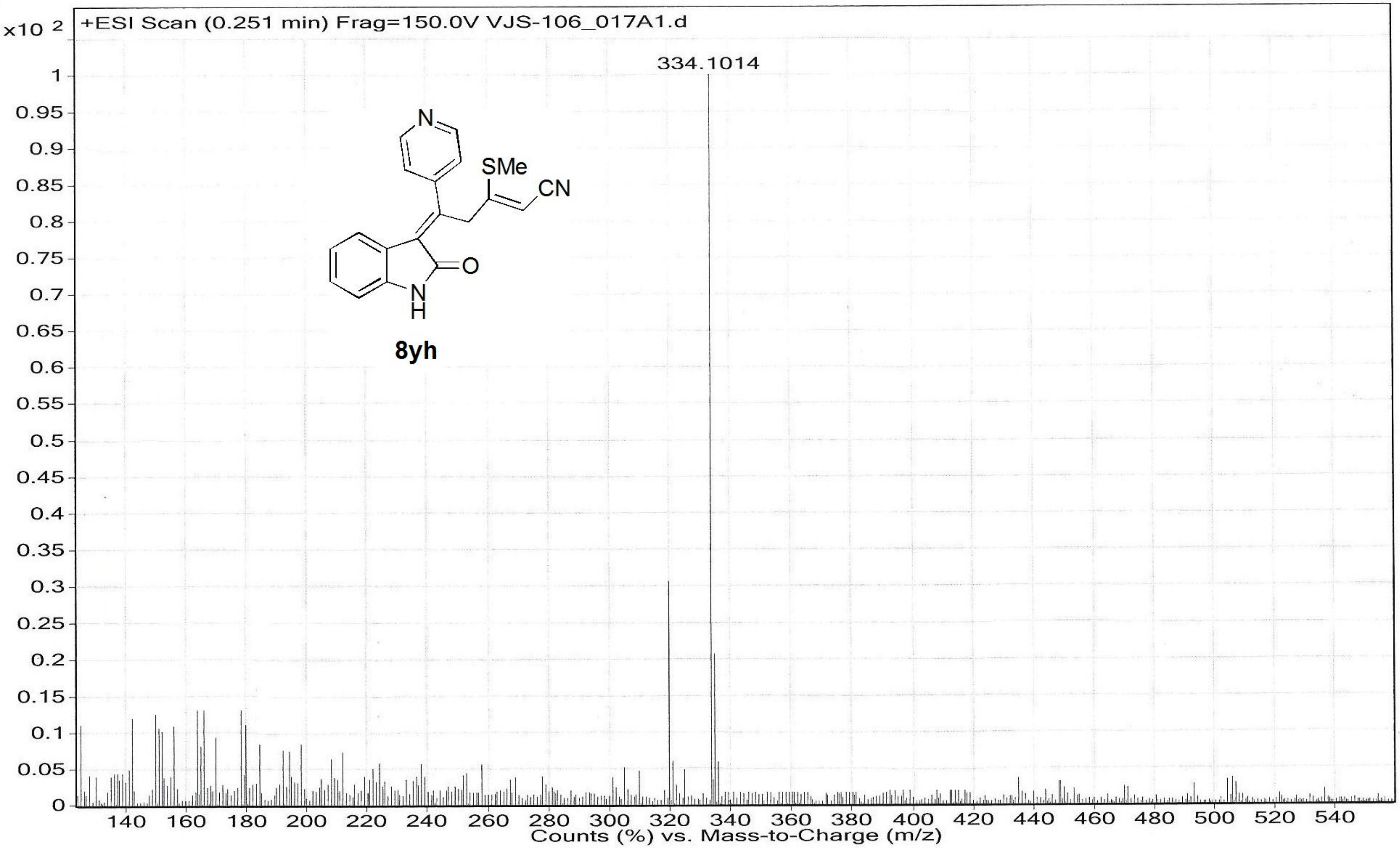
x10 2
+ESI Scan (0.251 min) Frag=150.0V VJS-106_017A1.d
334.1014
SMe
CN
N
O
H
8yh
1
0.95
0.9
0.85
0.8
0.75
0.7
0.65
0.6
0.55
0.5
0.45
0.4
0.35
0.3
0.25
0.2
0.15
0.1
0.05
0
140 160 180 200 220 240 260 280 300 320 340 360 380 400 420 440 460 480 500 520 540
Counts (%) vs. Mass-to-Charge (m/z)

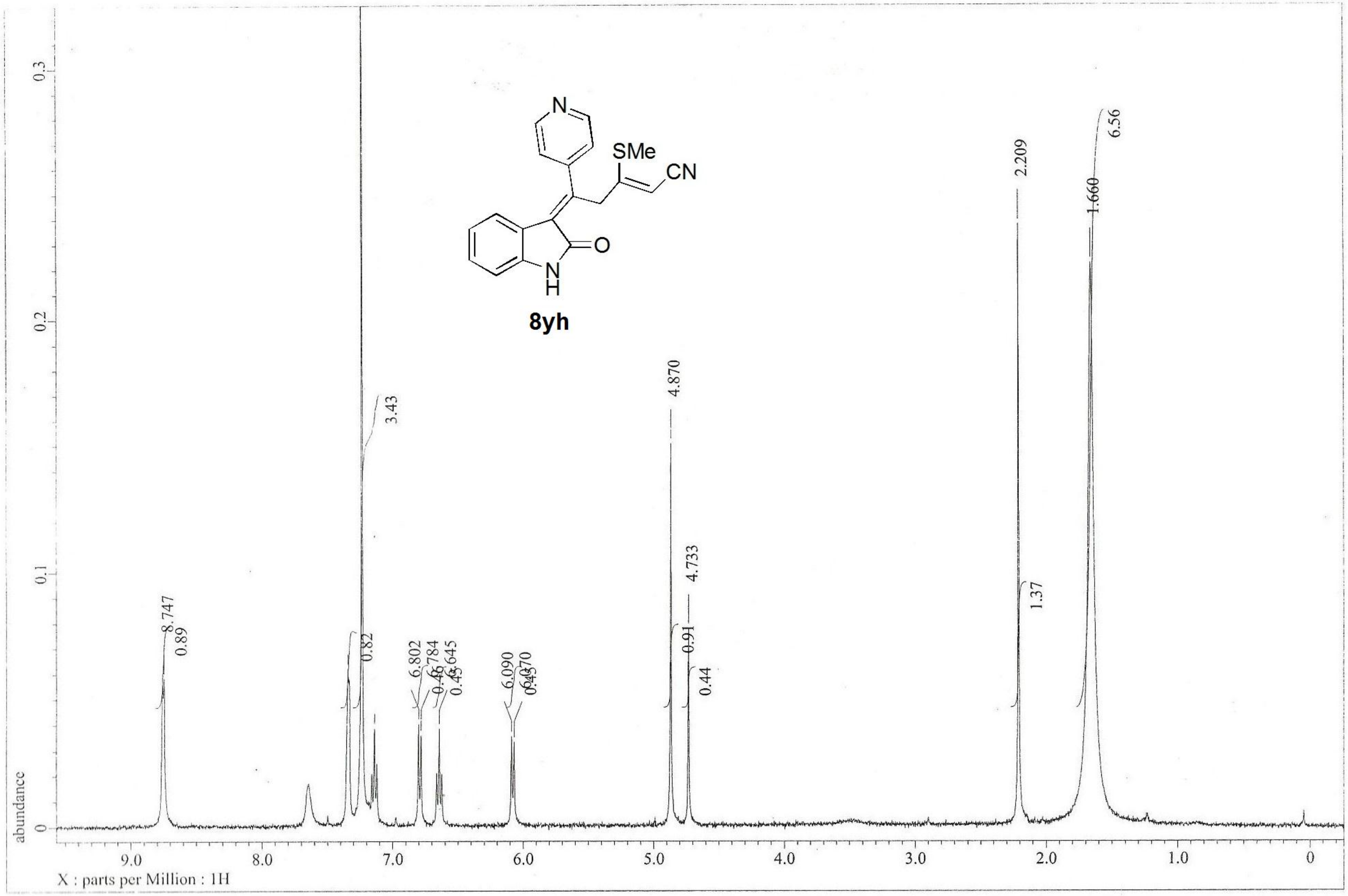

8yh
X : parts per Million : 1H
abundance

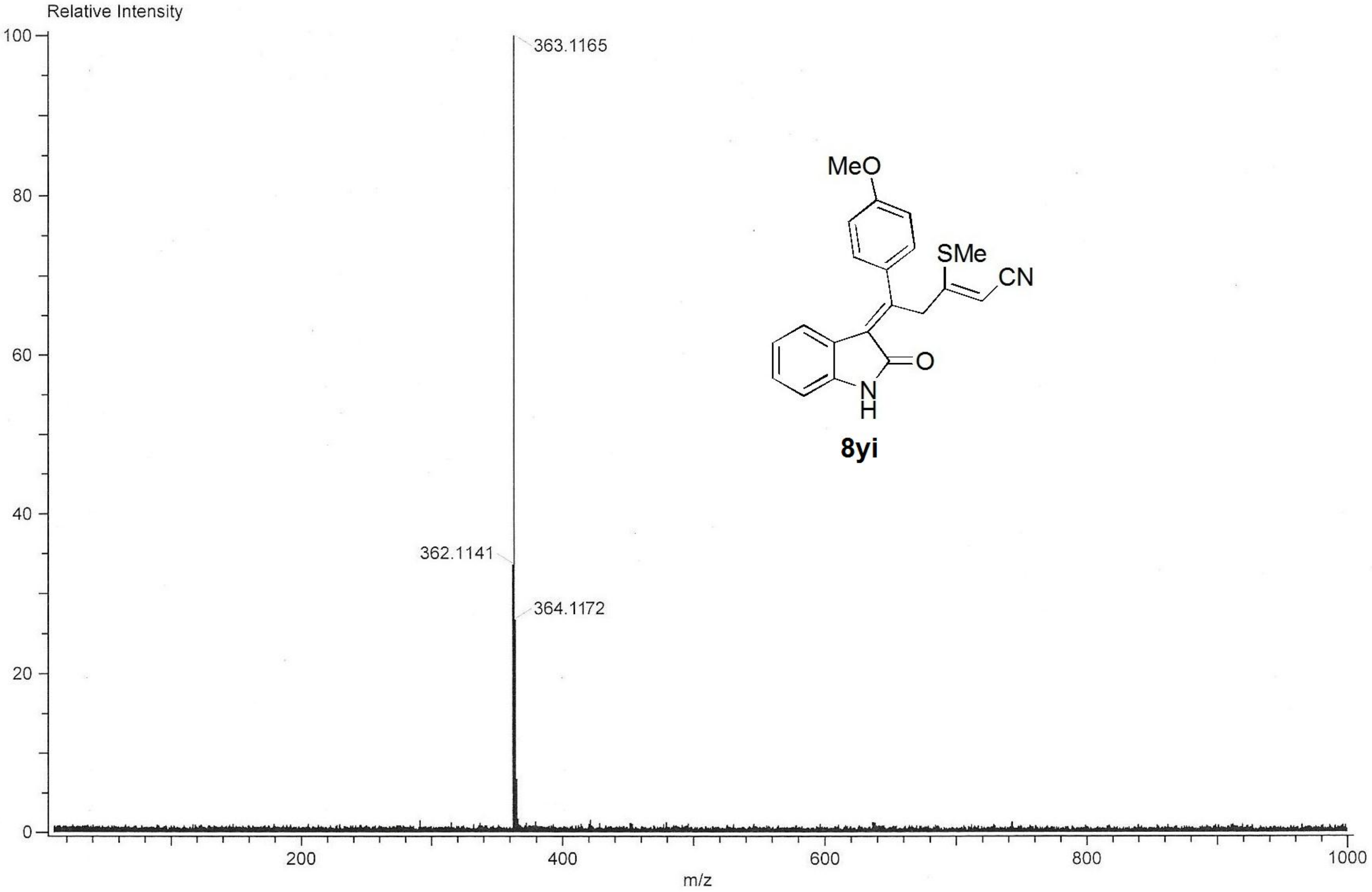

Relative Intensity
100
80
60
40
20
0
363.1165
362.1141
364.1172
200
400
600
800
1000
m/z
MeO
SMe
CN
O
N
H
8yi

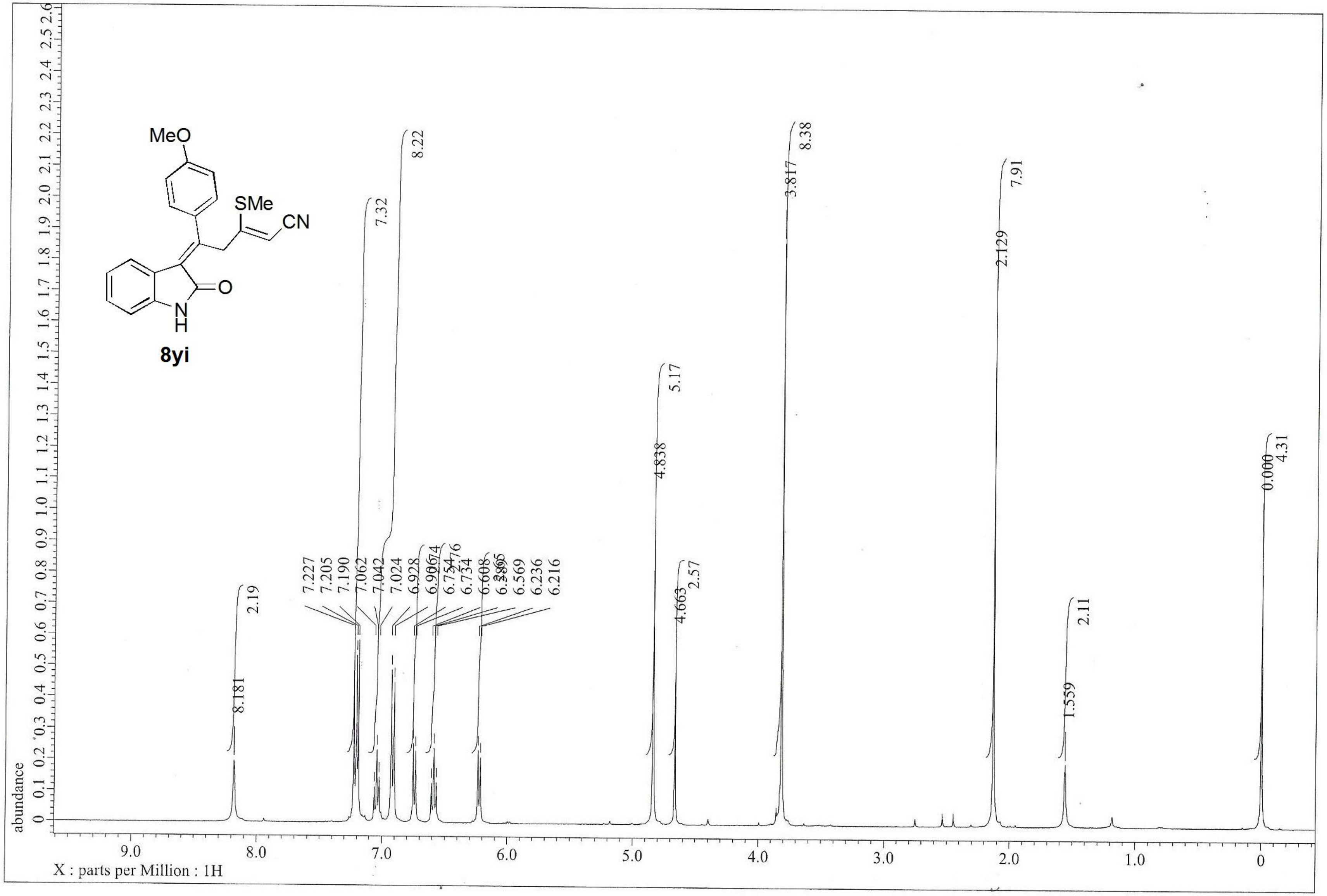
MeO
SMe
CN
O
N
H
8yi
abundance
X : parts per Million : 1H
2.19
8.181
7.227
7.205
7.190
7.062
7.042
7.024
6.928
6.734
6.608
6.569
6.236
6.216
7.32
8.22
5.17
4.838
2.57
4.663
8.38
3.817
7.91
2.129
2.11
1.559
4.31
0.000

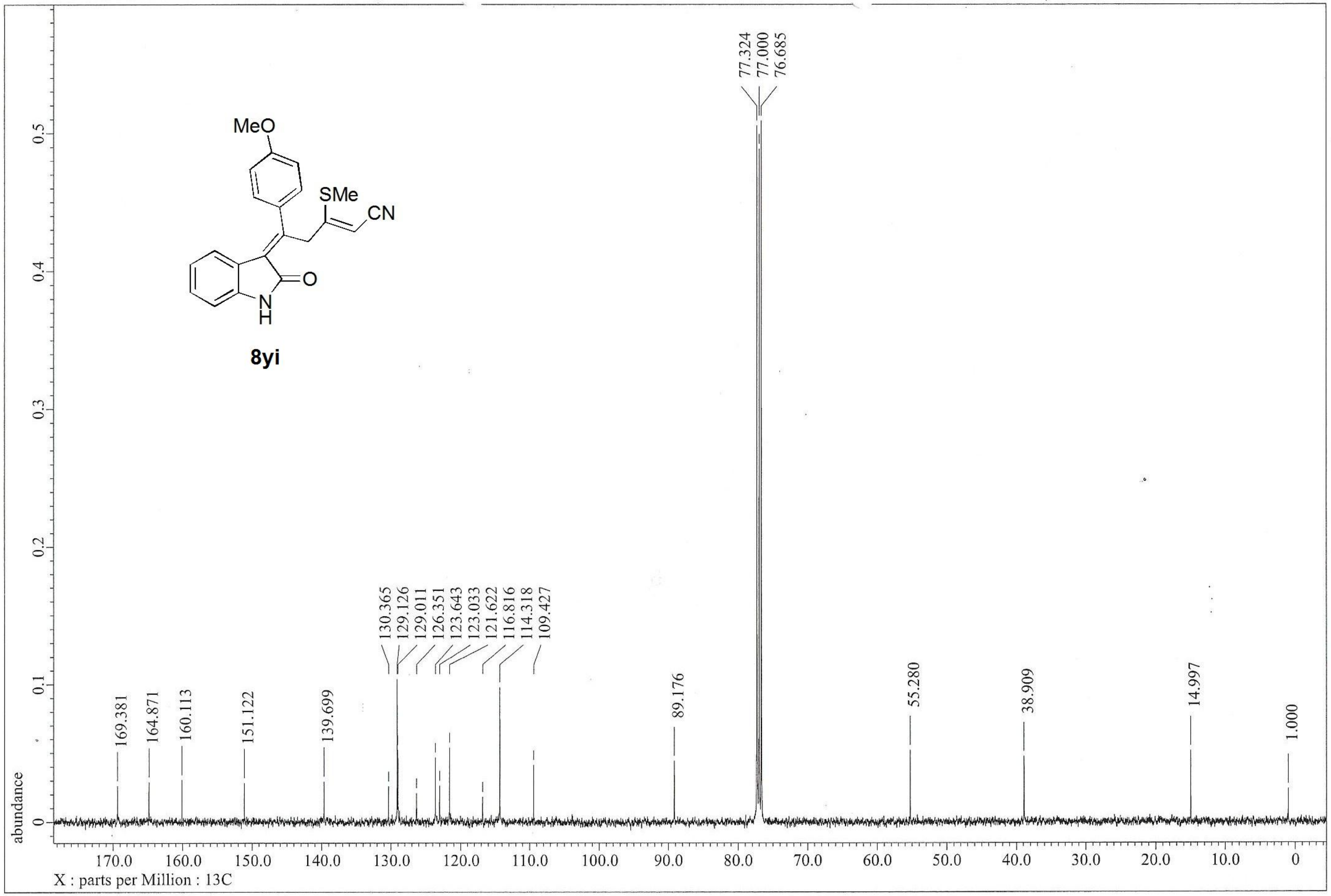
MeO
SMe
CN
O
N
H
8yi
77.324
77.000
76.685
130.365
129.126
129.011
126.351
123.643
123.033
121.622
116.816
114.318
109.427
169.381
164.871
160.113
151.122
139.699
89.176
55.280
38.909
14.997
1.000
abundance
X : parts per Million : 13C

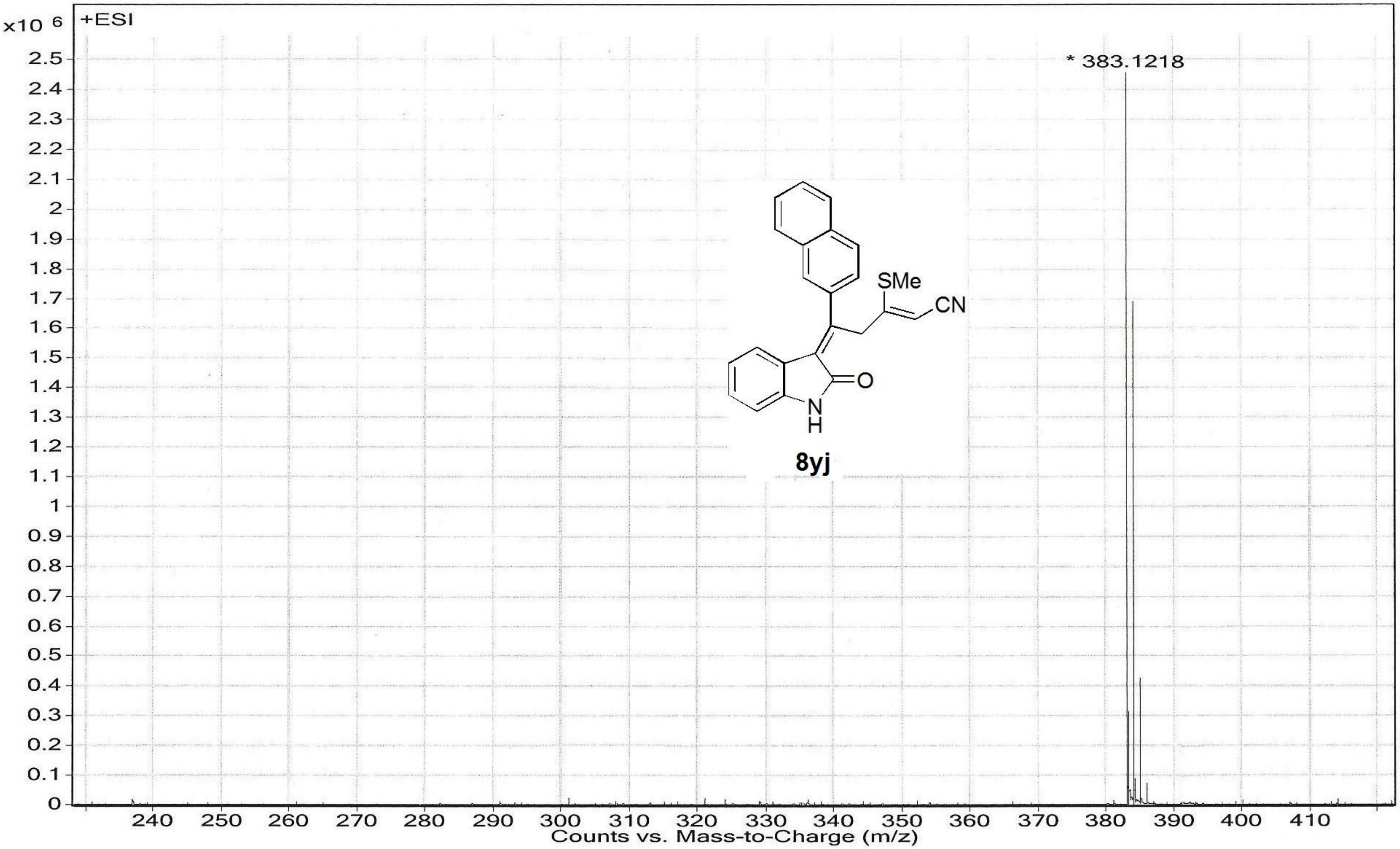
x10 6
+ESI
* 383.1218
SMe
CN
O
N
H
8yj
Counts vs. Mass-to-Charge (m/z)

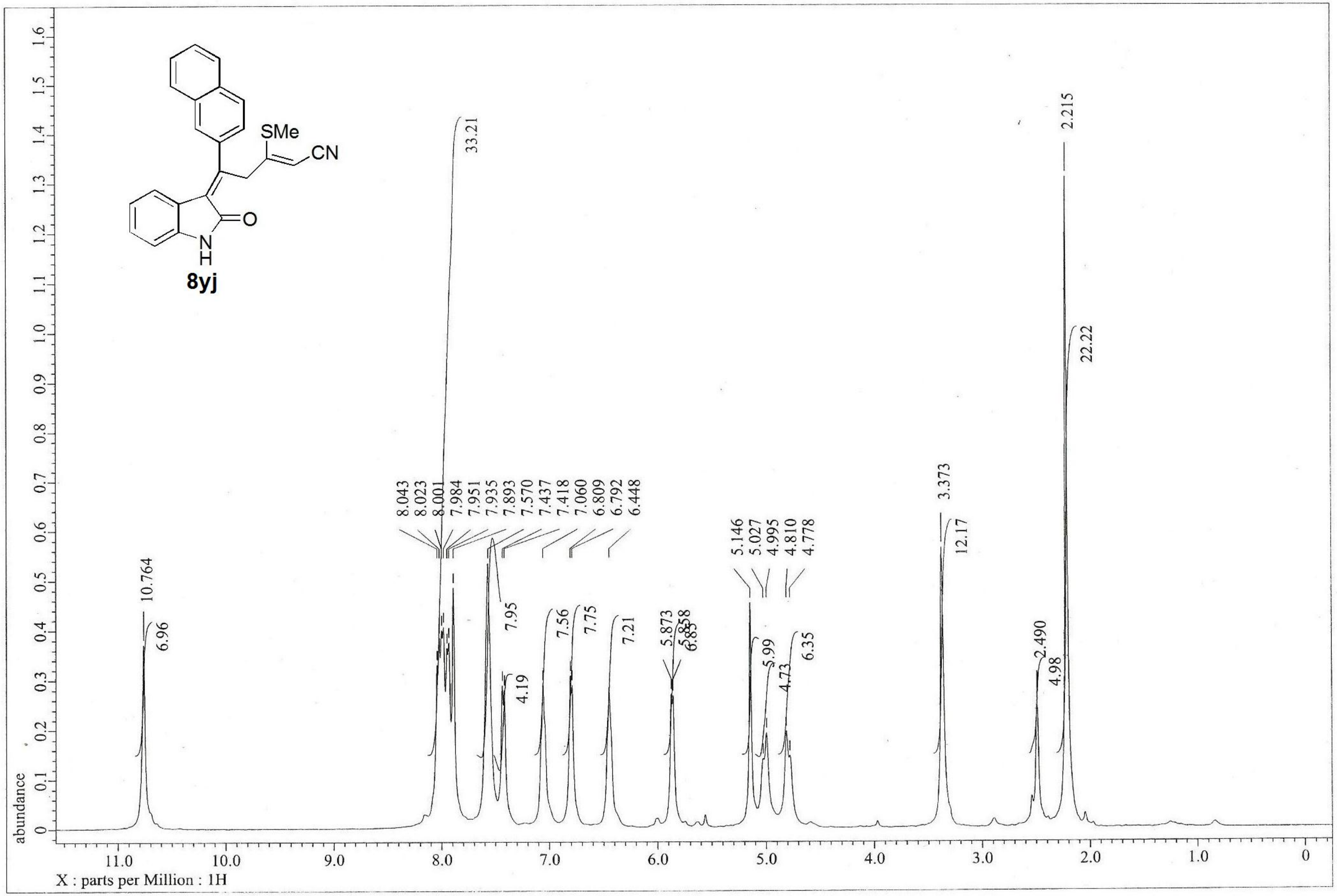
SMe
CN
O
N
H
8yj
10.764
6.96
8.043
8.023
8.001
7.984
7.951
7.935
7.893
7.570
7.437
7.418
7.060
6.809
6.792
6.448
33.21
7.95
4.19
7.56
7.75
7.21
5.873
5.858
6.85
5.146
5.027
4.995
4.810
4.778
5.99
4.73
6.35
3.373
12.17
2.490
4.98
2.215
22.22
abundance
X : parts per Million : 1H

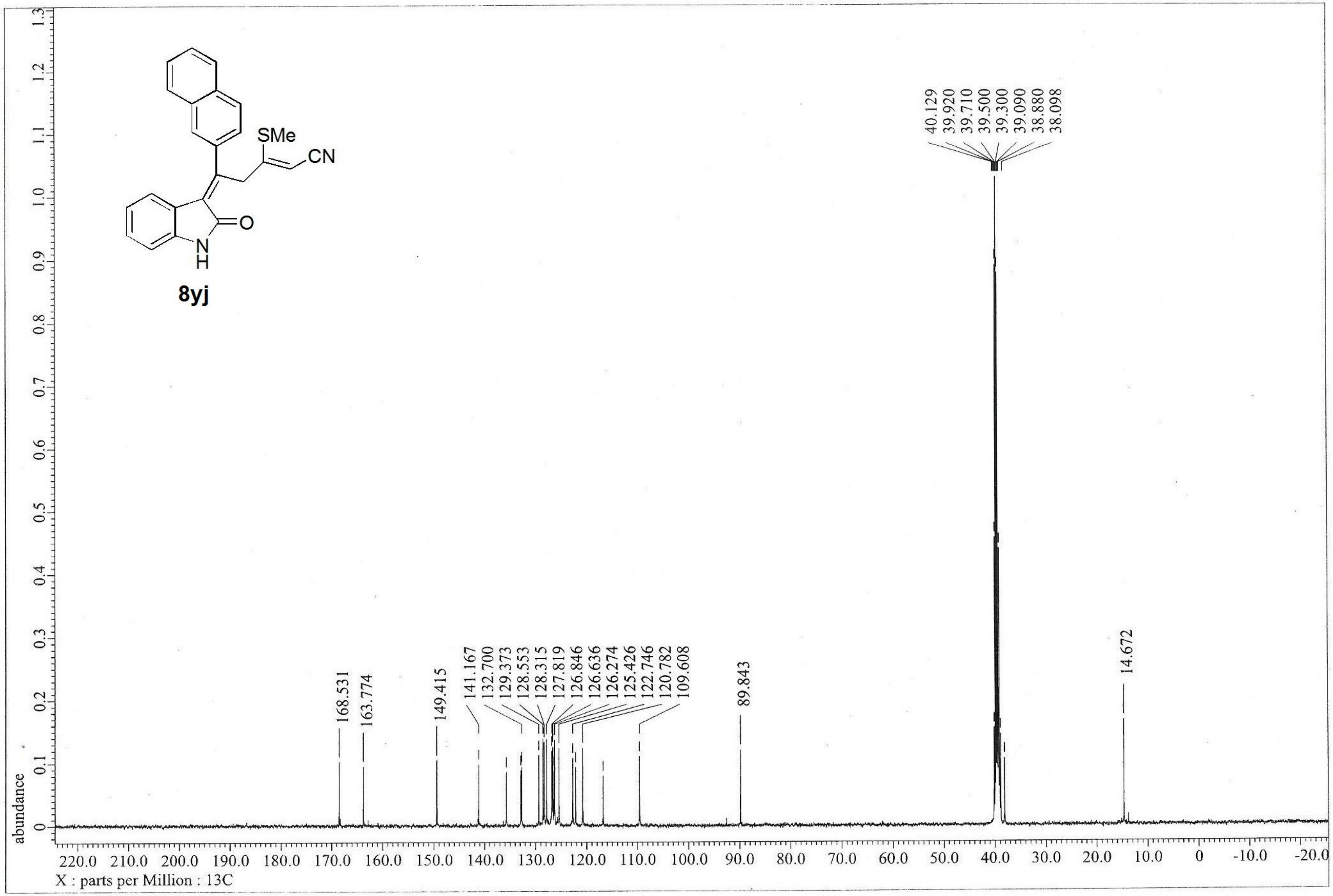
SMe
CN
N
H
O
8yj
40.129
39.920
39.710
39.500
39.300
39.090
38.880
38.098
168.531
163.774
149.415
141.167
132.700
129.373
128.553
128.315
127.819
126.846
126.636
126.274
125.426
122.746
120.782
109.608
89.843
14.672
abundance
X : parts per Million : 13C

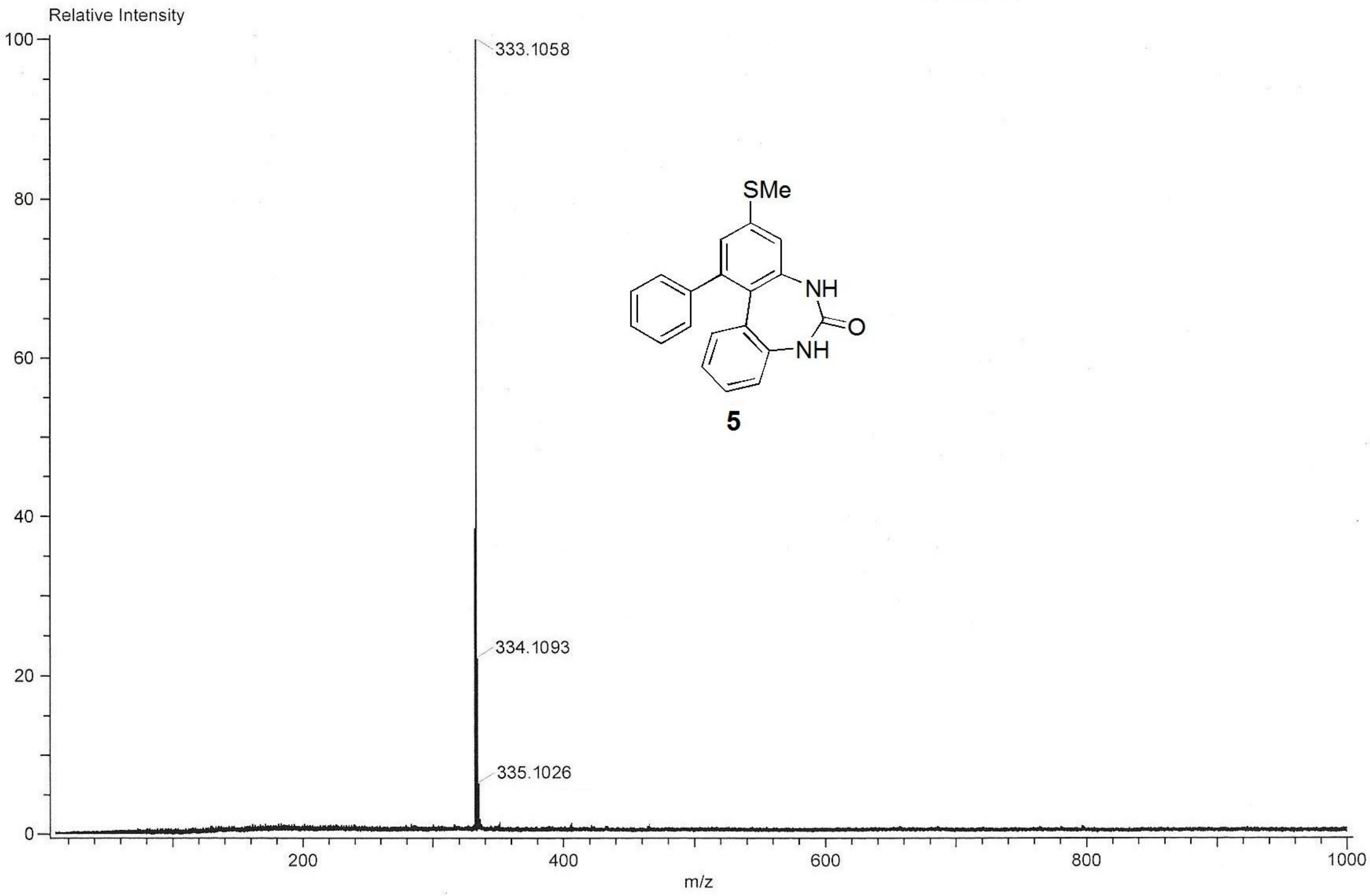
Relative Intensity
100
80
60
40
20
0
333.1058
334.1093
335.1026
200
400
600
800
1000
m/z
SMe
NH
O
NH
5

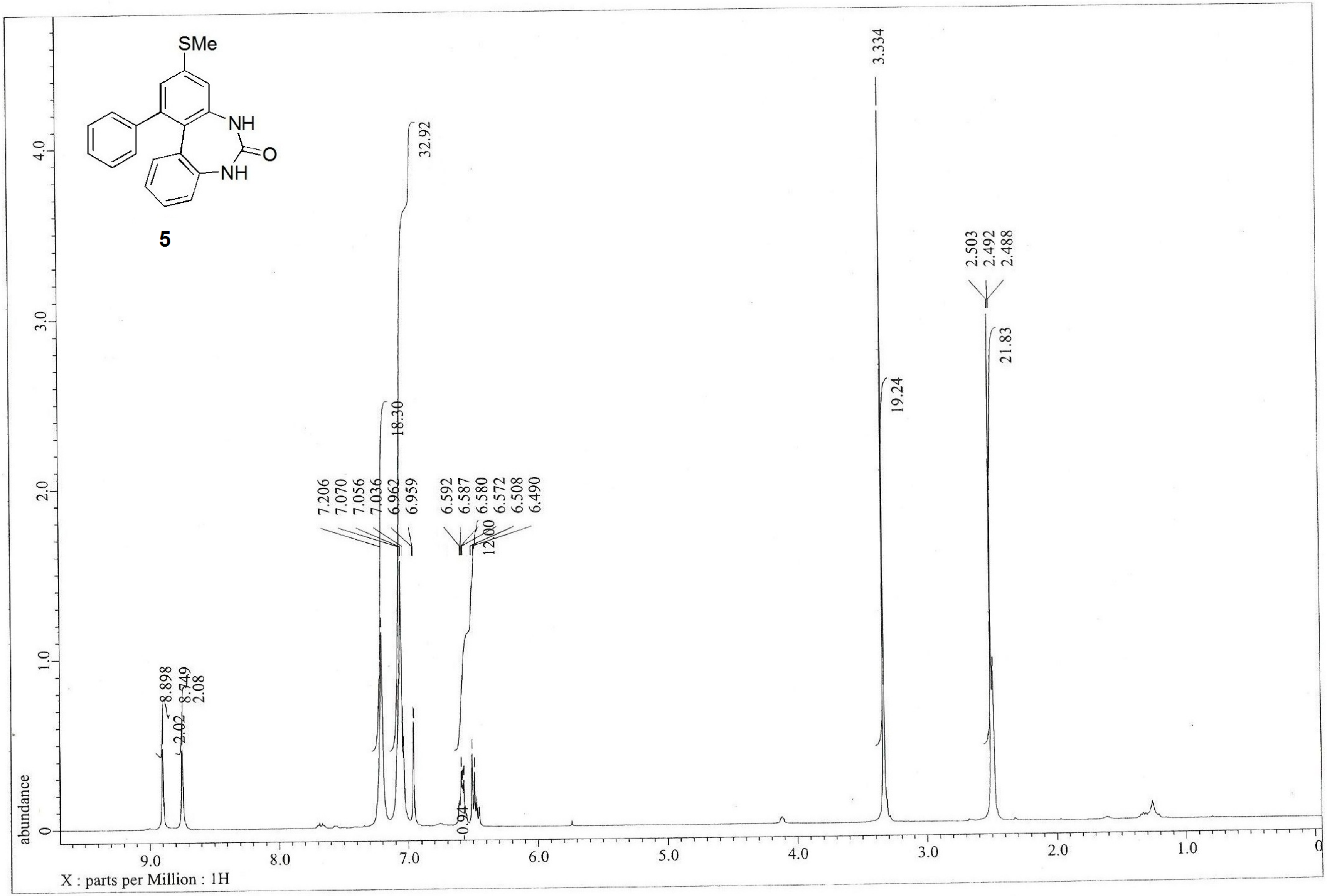
SMe
NH
O
NH
5
abundance
4.0
3.0
2.0
1.0
0
9.0
8.0
7.0
6.0
5.0
4.0
3.0
2.0
1.0
0
X : parts per Million : 1H
8.898
8.749
2.08
2.02
7.206
7.070
7.056
7.036
6.962
6.959
18.30
32.92
6.592
6.587
6.580
6.572
6.508
6.490
12.00
0.94
3.334
19.24
2.503
2.492
2.488
21.83

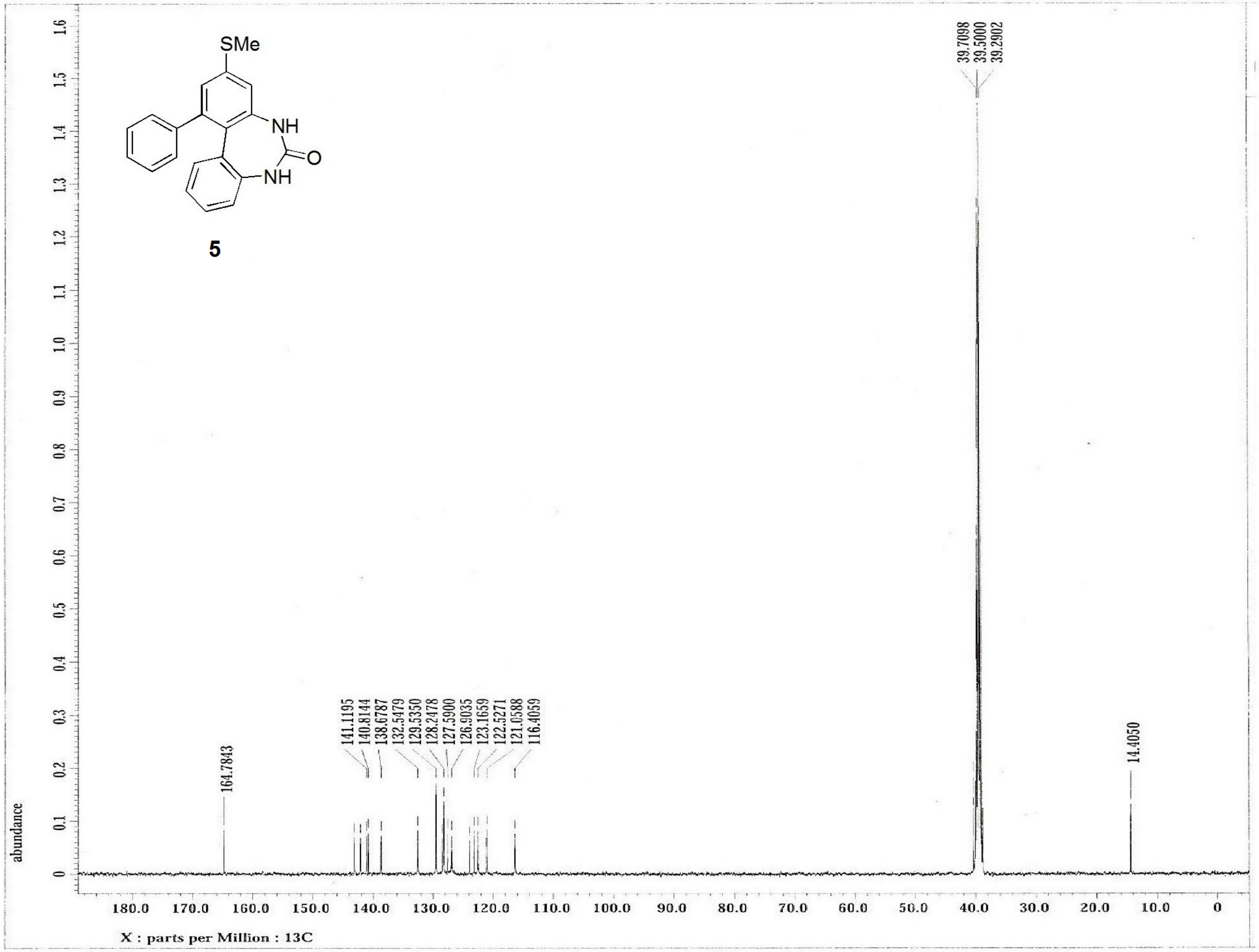
SMe
NH
O
NH
5
164.7843
141.1195
140.8144
138.6787
132.5479
129.5350
128.2478
127.5900
126.9035
123.1659
122.5271
121.0588
116.4059
39.7098
39.5000
39.2902
14.4050
abundance
X : parts per Million : 13C

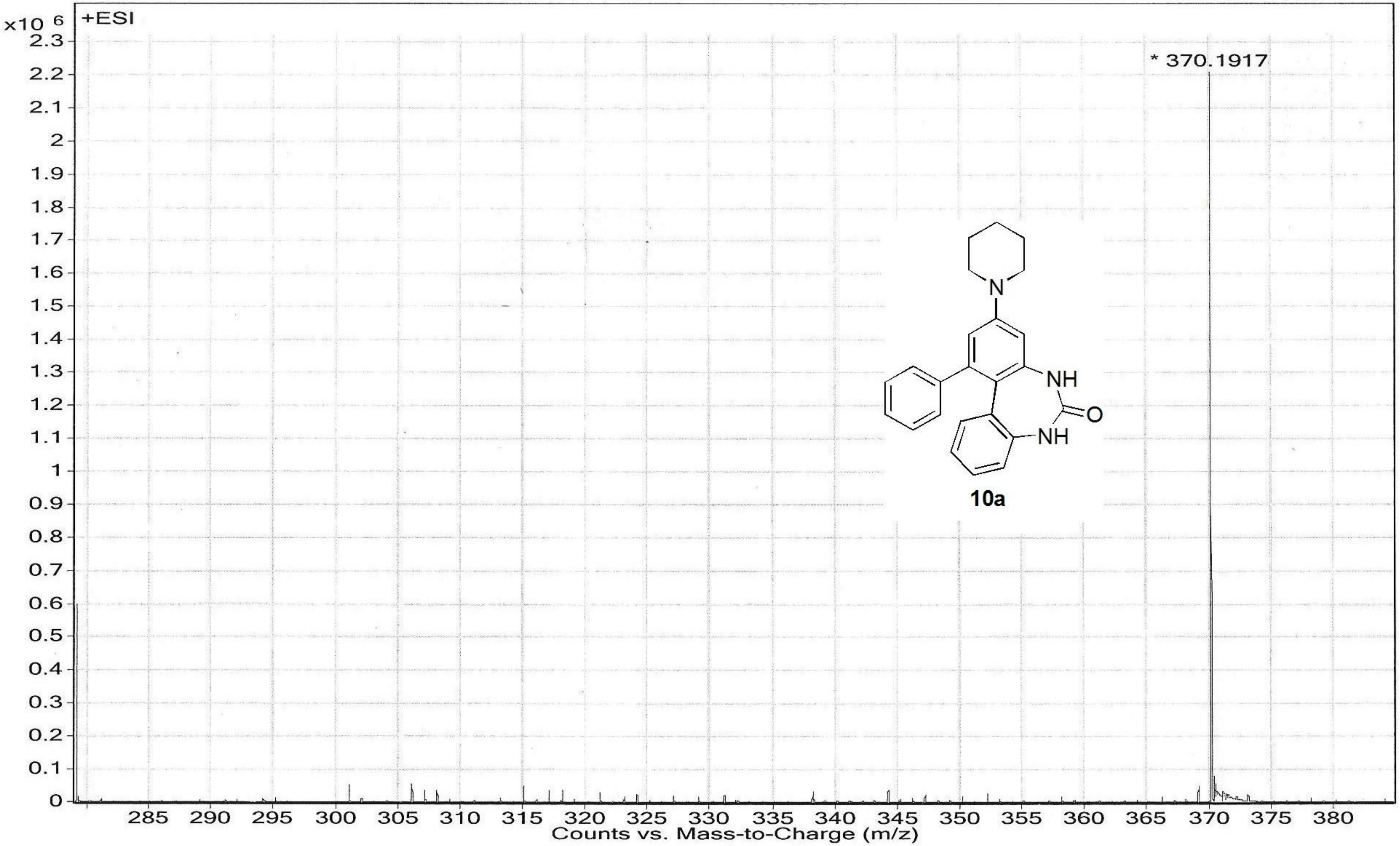

x10 6
+ESI
* 370.1917
NH
O
NH
N
10a
Counts vs. Mass-to-Charge (m/z)

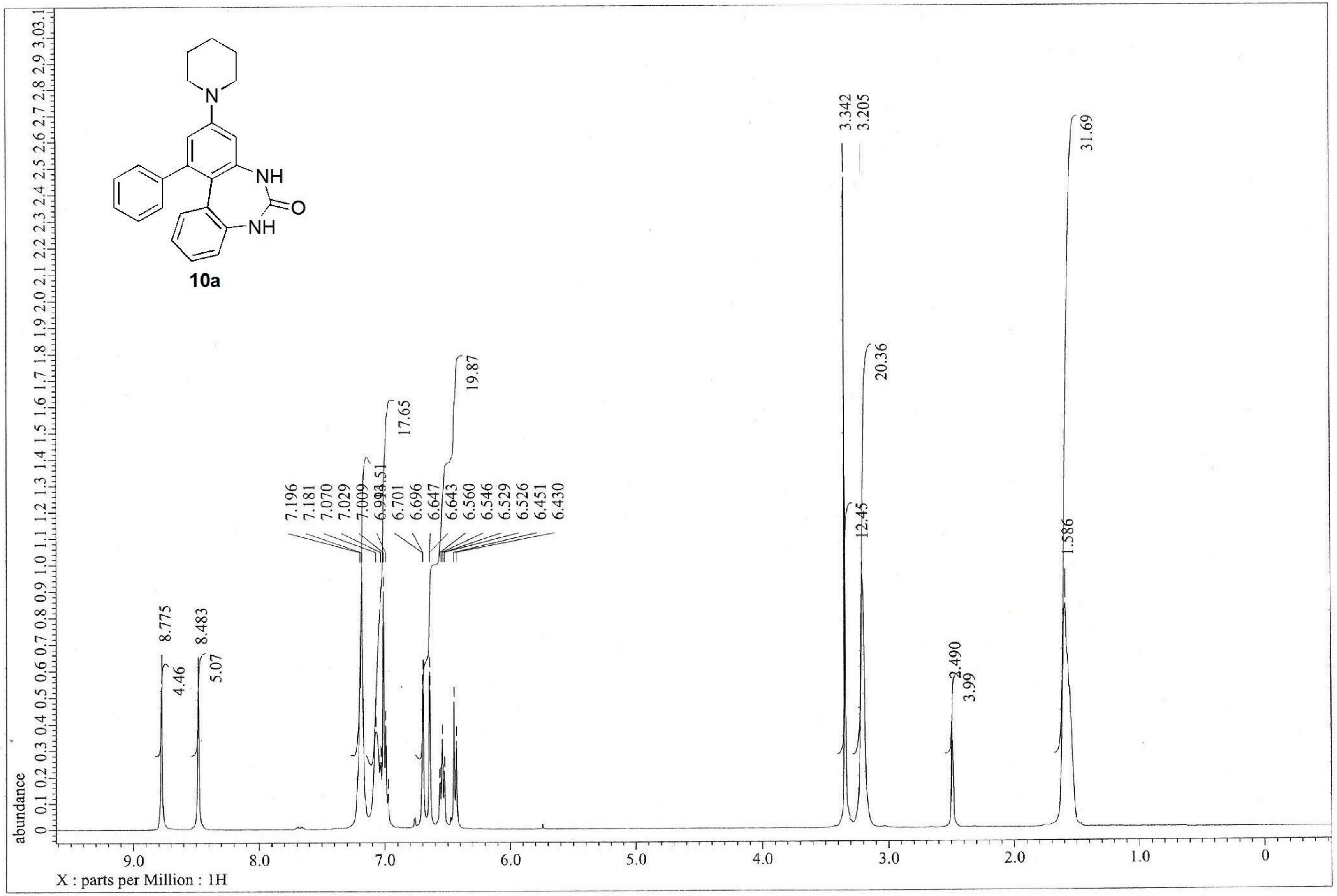
10a
NH
O
NH
N
8.775
8.483
4.46
5.07
7.196
7.181
7.070
7.029
7.009
6.701
6.696
6.647
6.643
6.560
6.546
6.529
6.526
6.451
6.430
17.65
19.87
3.342
3.205
12.45
20.36
2.490
3.99
1.586
31.69
abundance
X : parts per Million : 1H
9.0
8.0
7.0
6.0
5.0
4.0
3.0
2.0
1.0
0

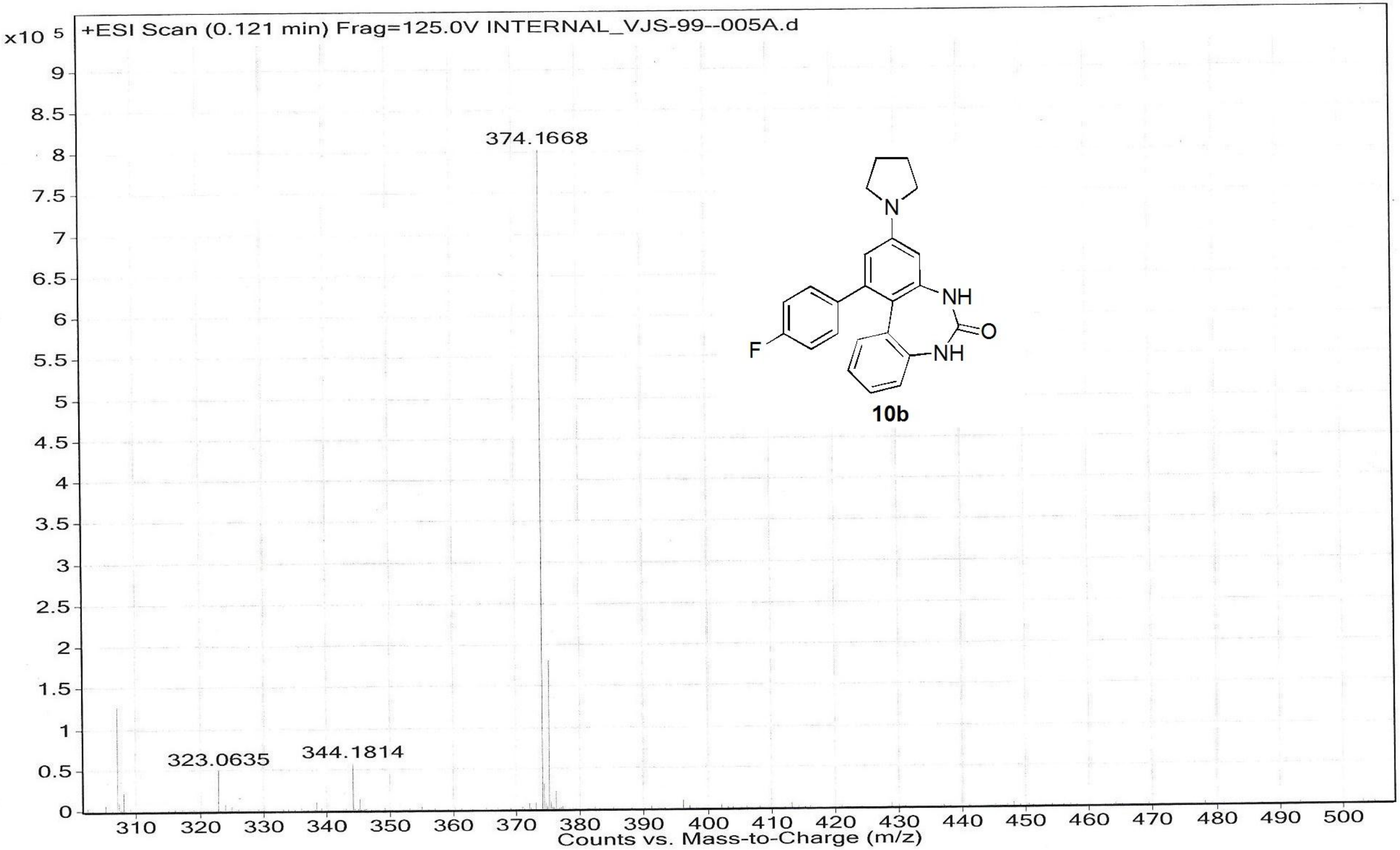
x10 5
+ESI Scan (0.121 min) Frag=125.0V INTERNAL_VJS-99--005A.d
9
8.5
8
7.5
7
6.5
6
5.5
5
4.5
4
3.5
3
2.5
2
1.5
1
0.5
0
374.1668
323.0635
344.1814
310 320 330 340 350 360 370 380 390 400 410 420 430 440 450 460 470 480 490 500
Counts vs. Mass-to-Charge (m/z)
N
NH
O
NH
F
10b

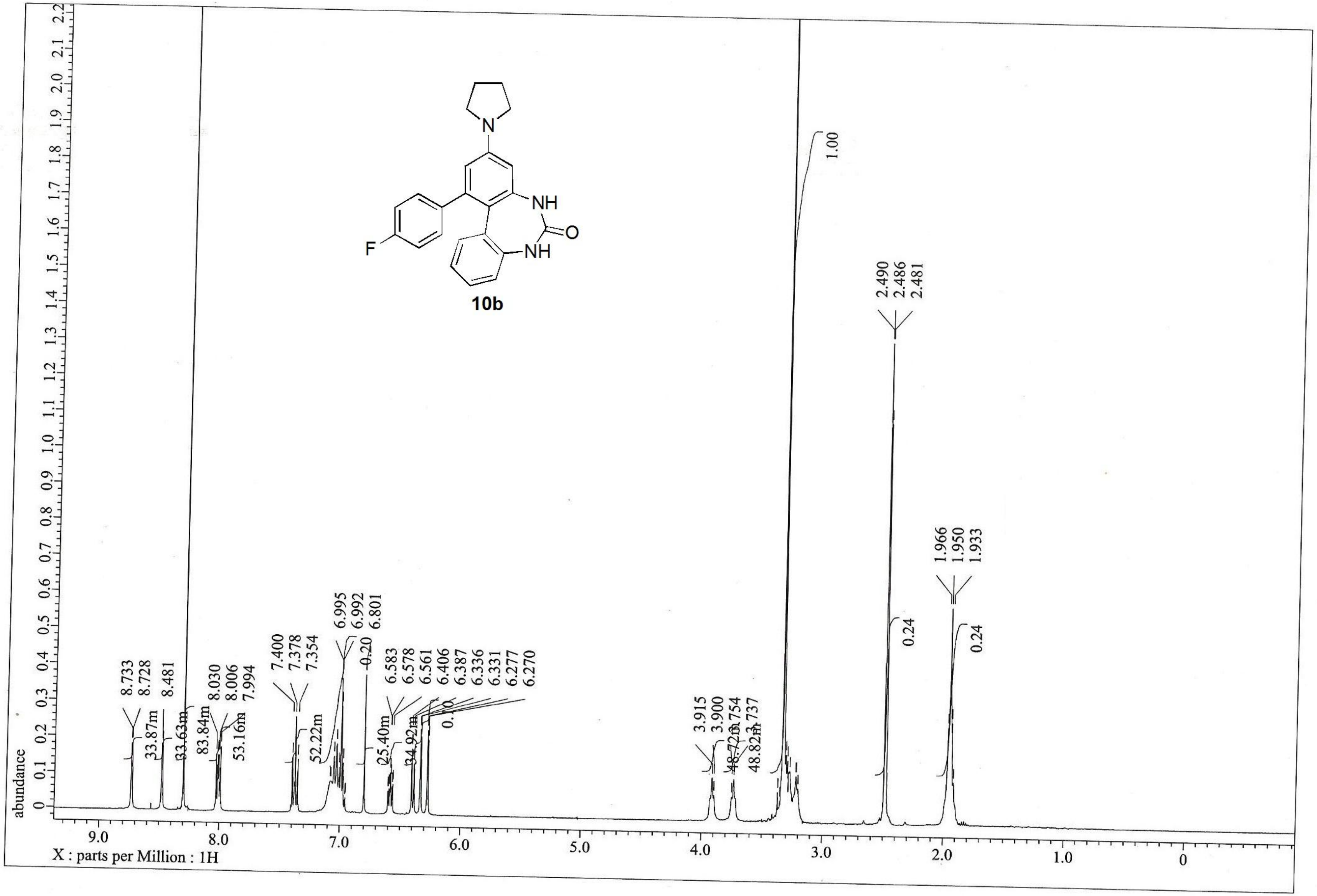

10b
NH
O
NH
N
F
1.00
2.490
2.486
2.481
0.24
1.966
1.950
1.933
0.24
8.733
8.728
8.481
8.030
8.006
7.994
7.400
7.378
7.354
6.995
6.992
6.801
6.583
6.578
6.561
6.406
6.387
6.336
6.331
6.277
6.270
3.915
3.900
3.754
3.737
abundance
X : parts per Million : 1H
